# Bacterial Quorum-Sensing Molecules as Promising Natural Inhibitors of *Candida albicans* Virulence Dimorphism: An *In Silico* and *In Vitro* Study

**DOI:** 10.3389/fcimb.2021.781790

**Published:** 2021-12-03

**Authors:** Ravi Jothi, Nagaiah Hari Prasath, Shanmugaraj Gowrishankar, Shunmugiah Karutha Pandian

**Affiliations:** Department of Biotechnology, Alagappa University, Karaikudi, India

**Keywords:** quorum-sensing molecules, farnesol, *C. albicans*, diketopiperazines, dimorphism, antihyphal, *in silico* docking and molecular dynamic simulation

## Abstract

Farnesol, a self-secreted quorum-sensing molecule (QSM) of *Candida albicans*, has been known to limit yeast-to-hyphal transition by blocking the RAS1–cAMP–PKA pathway. In a similar fashion, certain bacterial QSMs have also been reported to be successful in attenuating *C. albicans* biofilm and hyphal formation at relatively high cell density. This prompted us to investigate the antihyphal efficacy of certain bacterial QSMs through virtual docking against seminal drug targets, *viz*., CYCc and RAS1, that have been reported to be the hallmark players in *C. albicans* dimorphic virulence cascade. Against this backdrop, 64 QSMs belonging to five different bacterial QS signaling systems were subjected to initial virtual screening with farnesol as reference. Data of the virtual screening unveiled QSMs belonging to diketopiperazines (DKPs), i.e., 3-benzyl-6-isobutylidene-2,5-piperazinedione (QSSM 1157) and cyclo(l-Pro-l-Leu) (QSSM 1112), as potential inhibitors of CYCc and RAS1 with binding energies of −8.2 and −7.3 kcal mol^−1^, respectively. Further, the molecular dynamics simulations (for 50 ns) of CYCc-QSSM 1157 and RAS1-QSSM 1112 complexes revealed the mean ligand root mean square deviation (RMSD) values of 0.35 and 0.27 Å, respectively, which endorsed the rigid nature, less fluctuation in binding stiffness, and conformation of binding complexes. Furthermore, the identified two QSMs were found to be good in solubility, absorption, and permeation and less toxic in nature, as revealed by pharmacokinetics and toxicity analyses. In addition, the *in vitro* antihyphal assays using liquid and solid media, germ-tube experiment, and microscopic analysis strongly validated DKP-QSSM 1112 as a promising inhibitor of hyphal transition. Taken together, the present study unequivocally proves that DKPs can be used as potent inhibitors of *C. albicans* virulence dimorphism.

## 1 Introduction

Despite the advances in modern medicine, the management of infectious diseases has become more challenging, as microbial pathogens consistently break down every antimicrobial wall through a phenomenon called “antimicrobial resistance” (Llor and Bjerrum, 2014; Dadgostar, 2019). At times, the situation becomes worse with fungus-associated infections owing to the limitation of therapeutic options (Roemer and Krysan, 2014). Among the various fungal species, *Candida albicans* is the most representative pathogen at clinical setup, which causes severe contagious infections in humans (Jha and Kumar, 2018). *C. albicans* is a diploid polymorphic fungus that asymptomatically colonizes various niches of healthy humans as commensal. However, given the opportunity, *C. albicans* turns into a pathogen and causes clinically diverse infections, especially in individuals with disturbed immune surveillance or other debilitating conditions (Garcia et al., 2021). Various endogenous and exogenous signals facilitate *C. albicans* to manifest its devastating pathogenesis. Nevertheless, a plethora of studies on *C. albicans* pathogenesis at the molecular level have reinforced the morphological transition from yeast to hyphae as the hallmark event, which in turn triggers all other virulence traits (at any level of pathogenic circumstances) in a cascade manner (Calderone and Fonzi, 2001; Banerjee et al., 2019). Therefore, identifying molecules with potency to target dimorphic switching has been considered as one of the promising alternatives to effectively combat the infections associated with antifungal-resistant *C. albicans* by nullifying the phenomenon of selection pressure.

To date, numerous natural/synthetic compounds have been reported for their remarkable antihyphal efficacy against *C. albicans* (Manoharan et al., 2017; Priya and Pandian, 2020). However, farnesol, being a self-produced quorum-sensing molecule (QSM) of *C. albicans*, creates a high level of curiosity due to its unique mode of action. The presence of QSM in *C. albicans* was first reported by Hornby et al. (Hornby et al., 2001), and they made it clear that besides external signal, internally secreted farnesol also modulates the regulation of yeast-to-hyphal transition and subsequent biofilm formation in accordance with cell density. After the discovery of farnesol, few other research groups have tried to find out its accurate mode of action in inhibiting yeast-to-hyphal transitions (Nickerson and Atkin, 2017; Chen et al., 2018), but a clear understanding is still lacking. It has been well studied and reported that farnesol negatively regulates the RAS1–cAMP–PKA pathway by hindering the functions of CYR1 by binding with one of its active domains called CYCc (Davis-Hanna et al., 2008; Hall et al., 2011). The GTP-bound RAS1 (activated) directly interacts with CYR1 to catalyze the synthesis of cAMP that eventually activates PKA leading to the expression of several virulence genes in response to different environmental cues (Wang, 2013). Thus, the RAS1–cAMP–PKA pathway seems to be responsible for the expression of several genes associated with yeast-to-hyphal transition as well as other virulence traits and establishes infection (Wang, 2013). Furthermore, farnesol produced by *in situ* planktonic cells also limits the *C. albicans* biofilm formation owing to the strong association between hyphal morphology and biofilm development (Ramage et al., 2002). Apart from the fungal world, farnesol has also been shown to thwart the virulence such as biofilm and lipase productions in bacteria, *viz*., *Staphylococcus aureus*, and sensitizes the drug-resistant *S. aureus* to gentamicin (Jabra-Rizk et al., 2006).

Given the prominence of farnesol in effectively interfering with the yeast-to-hyphal switch, several natural and synthetic farnesol analogs have been investigated for their improved inhibitory efficacy against *C. albicans* dimorphic switching (Shchepin et al., 2003; Lee et al., 2020). Following the same paradigm, certain QSMs of bacteria have been envisaged and well demonstrated to greatly influence the morphogenesis of *C. albicans*. For instance, the QSMs *N*-(3-oxododecanoyl)-l-homoserine lactone (3OC12HSL), *cis*-2-dodecenoic acid (BDSF), and *trans*-2-decenoic acid (SDSF) produced by *Pseudomonas aeruginosa*, *Burkholderia cenocepacia*, and *Streptococcus mutans*, respectively, have been reported to substantially limit *C. albicans* hyphae without altering its basic metabolism (Grainha et al., 2020). Besides, it has also been deciphered that 3OC12HSL interferes with the *C. albicans* yeast-to-hyphal transition by mimicking the function of farnesol. The other two QSMs, namely, BDSF and SDSF, have been reported to follow a distinct mode of action unlike 3OC12HSL (Hogan et al., 2004; Grainha et al., 2020). Naturally, such antagonistic interactions between bacteria and fungi occur in human microbiota wherein the bacterial QSMs play an indispensable role in controlling fungal growth to maintain the ecological balance. Therefore, understanding the mighty role of bacterial QSMs, it is envisaged that these bacterial QSMs could be an effective agent to probe against fungal filamentation by mimicking the action mechanism of the farnesol molecule.

Against this backdrop, the present study was focused to screen and envisage bacterial QSMs with potency to inhibit the seminal protein targets of *C. albicans* filamentation through *in silico* and *in vitro* analyses. Owing to the essentiality of CYR1 and RAS1 in farnesol-mediated hyphal inhibition, they were chosen as potential drug targets in the current study (Wang, 2013; Boutet et al., 2016). Overall, the current study is the first of its kind that provides potential insights into bacterial QSMs mediated inter-microbial cross-talk. Further, the outcome of the study will prompt the researchers working in the arena of alternative medicine to refocus/revisit the bacterial QSMs as a novel hyphal inhibitor against pathogenic fungi.

## 2 Materials and Methods

### 2.1 Homology Modeling

The experimentally solved crystal structure of CYCc and RAS1 was not publicly available in the Protein Data Bank (PDB). Therefore, three-dimensional (3D) structures of these proteins were modeled on the basis of homology modeling using SWISS-Model (https://swissmodel.expasy.org/). Initially, the amino acid sequences of these target proteins, i.e., CYCc (UniProt ID. P0CY32-1) and RAS1 (UniProt ID. A0A1D8PR83-1; aa length. 1,329–1,466), were retrieved from UniProt KnowledgeBase (UniProtKB) in FASTA format. The resulting sequence was submitted to PDB-BLAST in order to identify the appropriate templates for structural modeling. Finally, the 3D structures of CYCs and RAS1 were generated using SWISS-Model (Boutet et al., 2016).

### 2.2 Validation of Modeled Protein

The stereochemical qualities and reliability of modeled structures were verified by PROCHECK (Program to check the stereochemical quality of protein structures), a program that relies on the Ramachandran plot for structure validation. The number of factors such as overall G-factor, the total number of amino acid residues in the core, and allowed, generously allowed, and disallowed regions were considered for choosing the best model (Laskowski et al., 2005).

### 2.3 Protein Preparation

Further, the validated modeled structures of CYCs and RAS1 were prepared for docking using AutoDock. In order to minimize the energy, the solvated structures were refined through the computation of Gasteiger charges with the addition of the polar hydrogens. Finally, refined protein structures were subjected to further analysis.

### 2.4 Ligand Selection and Preparation

A total of 64 bacterial QSMs screened in this study were retrieved from the SigMol database created by Rajput et al. (2016) (http://bioinfo.imtech.res.in/manojk/sigmol) (Rajput et al., 2016). Basic information about the ligands such as molecular weight and 3D structure (.SDF) was retrieved from PubChem and ChemSpider databases, respectively. Furthermore, the AutoDock formats (.pdbqt) were generated through AutoDock Vina in PyRx 0.8 software for the molecular docking simulation (Rolta et al., 2020).

### 2.5 Docking-Based Virtual Screening

In order to identify the potential inhibitors against putative active sites of target proteins CYCc and RAS1, virtual screening was performed using PyRx, 0.8. The minimized proteins and ligand molecules were imported to PyRx, and blind docking was performed (Schuttelkopf and van Aalten, 2004). The full grid map was constructed around the complete target proteins. The resulting docking conformations were ranked based on their binding affinities in comparison with the reference molecule (farnesol). Finally, the best-docked complexes were visualized using Discovery studio visualize and ligplot+.

### 2.6 Molecular Dynamics Simulation

The best interactive ligand molecules with their target proteins were subjected to 50 ns of molecular dynamics (MD) simulation by Gromacs 5.1.4 simulation package (http://www.gromacs.org/) using all-atom optimized potentials for liquid simulations (OPLS-AA) force field (Abdulazeez, 2019). The topological parameter files of proteins and ligand molecules were prepared from GROMOS96 force field and PRODRG2 web server, respectively. Initially, docked complexes were kept in a cubic box surrounded by SPC, a water molecule (water density 1.0) using SPC/E water models, and further, the whole system was neutralized by the addition of Na^+^ ions. The solvated structures were energy minimized using GROMOS54a7 force field and equilibrated by running simulations for 100 ps under constant NVT (300 K temperature) and NPT (1 atm pressure) ensemble. The equilibrated complexes were further extended to run production MD simulation for 50 ns at constant temperature and pressure. The GROMACS package was used to calculate the structural properties such as root mean square deviation (RMSD), root mean square fluctuation (RMSF), hydrogen bond radius of gyration, and solvent accessible surface area. Finally, the resulting values were plotted as graphs using Origin Pro.

### 2.7 Estimation of Pharmacokinetics and Toxicity Profile

The antihyphal inhibitors screened *via* molecular docking and molecular simulations were checked for various pharmacokinetic parameters using SwissADME online tool (http://www.swissadme.ch) (Rodrigues et al., 2020). The smileys of both ligands were collected from SigMol database and added into SwissADME as an input to analyze the principles of ADMET (absorption, distribution, metabolism, and elimination), pharmacokinetics, drug likeliness, and medical chemistry. In order to predict the toxicity dosage and toxicity class of the ligands, ProTox-II server (https://tox-new.charite.de/protox_II/) was utilized (Banerjee et al., 2018).

### 2.8 Strain and Culture Condition

The test organism *C. albicans* (ATCC 90028) used in the present study was procured from HiMedia, India. The fungus was maintained in Sabouraud Dextrose Agar (SDA) plates and routinely cultured in yeast extract peptone dextrose (YEPD) broth at 37°C until used for tests. The culture with 0.1 optical density (OD) (1 × 10^6^ CFU/ml) was used as the inoculum to perform antihyphal assays. The hyphal assay was performed in spider medium (consisting of mannitol 1%, K_2_HPO_4_ 0.2%, and nutrient broth 1%) to allow hyphal elongation.

### 2.9 *In Vitro* Filamentation Assay

In order to substantiate the *in silico* finding, *in vitro* antihyphal assay was performed using farnesol as the positive control (Priya and Pandian, 2020). For this experiment, one of the predicted ligands, i.e., QSSM 1112, was employed to assess its inhibitory propensity on *C. albicans* dimorphic switching. The QSSM 1112 (purity >98% based on high-performance liquid chromatograph) used in the current study was reported in our previous study (Gowrishankar et al., 2014) and deployed for assays by dissolving in methanol at the final concentration of 100 mg/ml. Briefly, *C. albicans* cells (1 × 10^6^ CFU/ml) were dispensed into 24-well microtiter plates (MTPs) containing 1 ml of spider medium supplemented with QSSM 1112 and farnesol at various concentrations (0–1,024 µg/ml) along with 10% of fetal bovine serum (FBS). After incubation at 37°C for 24 h, the pellets were resuspended in 10 µl of phosphate-buffered saline (PBS), and the ratio of yeast to hyphal cells was further confirmed by a light microscope at ×400 magnification (Nikon Eclipse 80i, Japan).

### 2.10 Germ-Tube Inhibition Assay

The efficiency of QSSM 1112 in inhibiting the *C. albicans* germ-tube formation was evaluated through germ-tube assay as described by Zhang et al. (2011) (Zhang et al., 2011). Here again, farnesol was used as a positive control for the comparative analysis. In brief, *C. albicans* cells (1 OD) were used to inoculate into the 200 µl of FBS along with QSSM 1112 at its hyphal inhibitory concentration (HIC), i.e., 64 µg/ml. The tubes were incubated at 37°C for 5 h to allow germ-tube induction. At different time intervals, i.e., 0, 2, 4, and 6 h, the cells were removed aseptically and examined under a light microscope at ×400 magnification in order to assess the germ-tube induction (Nikon Eclipse 80i, Japan).

Additionally, the inhibitory propensity of QSSM 1112 on preformed germ-tube formation was evaluated. Initially, the *C. albicans* cells were allowed for germ-tube induction following the protocol mentioned above. Then, the QSSM 1112 (at HIC) was manifested on germinated *C. albicans* cells and incubated at 37°C for 3 h. The cells without the active agent were considered as untreated control. Subsequent to incubation, the morphological changes between QSSM 1112-treated and untreated control cells were observed under a light microscope at ×400 magnification (Nikon Eclipse 80i, Japan).

### 2.11 Fluorescence Microscopic Analysis

To further ascertain the QSSM 1112 mediated hyphal and germ-tube inhibition, fluorescence microscopic analysis was performed. For hyphal assay, the *C. albicans* cells were allowed to form hyphal formation on a 1-cm^2^ glass slide in 24-well MTPs containing spider medium supplemented with farnesol and QSSM 1112 at their HIC. After incubation, non-adherent planktonic cells were removed, and adhered hyphal cells on the slides were stained with 1% acridine orange for 20 min.

For germ-tube inhibition assay, *C. albicans* cells were grown in test tubes containing spider medium supplemented with farnesol and QSSM 1112 at their HIC for 24 h. Subsequent to incubation, cell pellets were collected by centrifugation at 8,000 rpm for 10 min. Then, pellets were resuspended in 100 µl of PBS and followed by staining with 20 µl of 1% acridine orange for 20 min. After incubation, the difference in cell morphology was visualized under fluorescence microscopy (Ts2R, ECLIPSE, and Japan).

## 3 Results and Discussion

Clinical complications associated with the conventional antifungals immensely demand a new treatment strategy against the dimorphic fungus *C. albicans*. One of the proven strategies that propel in the arena of alternative medicine to get rid of drug resistance is “disarming the virulence without disturbing the metabolism” of any fungus (Charro and Mota, 2015). As hyphal transition is a hallmark event that lionizes commensal yeasts as opportunistic pathogens, blocking this morphological switch is imperative to control infections associated with *C. albicans* (Roemer and Krysan, 2014; Banerjee et al., 2019). So far, many molecules with antihyphal proficiency have been reported from natural and organic sources against *C. albicans* (Manoharan et al., 2017; Priya and Pandian, 2020). However, the QSMs of bacteria remain unexplored for antihyphal propensity. Few reports have been focused on the competitive antagonistic interactions between bacteria and *C. albicans*, which signify that bacterial QSMs could play a key role in targeting and blocking yeast-to-hyphal transition (Hogan et al., 2004; Nickerson and Atkin, 2017). This intertaxon chemical communication between bacteria and fungi prompted us to investigate the effect of bacterial QSMs as natural hyphal inhibitors against *C. albicans* through *in silico* approach. Therefore, in the current study, 64 QSMs belonging to five different bacterial QS signaling systems such as acylated homoserine lactones (AHLs), diketopiperazines (DKPs), diffusible signal factors (DSFs), autoinducer-2 (AI-2), and 4-hydroxy-2-alkylquinolines (HAQs) were selected and subjected to virtual docking against the two identified seminal drug targets (CYCc and RAS1) of *C. albicans*. It was anticipated that the outcome of the present study would render a basic understanding of the interaction of bacterial QSMs against important hyphal proteins of *C. albicans*.

### 3.1 Homology Modeling

Primarily, homology modeling of target protein structures was performed. Protein crystal structure is important for understanding the mechanisms of biological systems and protein complexes to manipulate or inhibit protein function (Vyas et al., 2012). Owing to the unavailability of resolved crystal structure for the target protein in PDB, computational-based techniques were used for 3D structure prediction. Homology modeling offers 3D models of protein structures when only sequence data are available (Satyanarayana et al., 2018).

**Figure 1:**
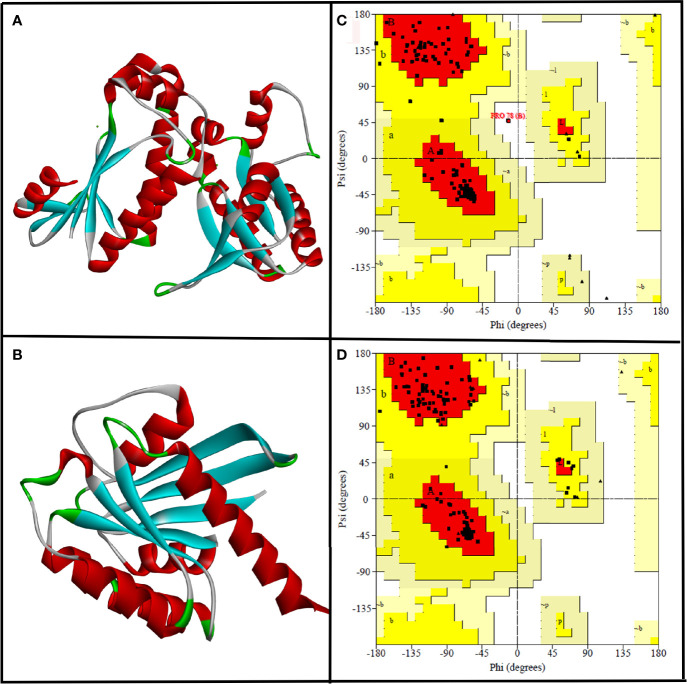
The three-dimensional structure of CYCc **(A)** and RAS1 **(B)** built using SWISS-MODEL. Ramachandran plot generated portraying the stereochemical qualities of the modeled CYCc **(C)** and RAS1 **(D)** proteins.

Since the experimentally solved 3D structure of selected target proteins, i.e., CYCc and RAS1, were not available in freely accessible public databases, homology modeling was done using SWISS-MODEL to obtain the good models. The primary sequences of target proteins CYCc (Q9P977_CANAX) and RAS1 (P0CY32_CANAX) were retrieved from UNIPROTKB database. Based on the results of SWISS-MODEL’s Critical Assessment of Structure Prediction (CASP), adenylate cyclase from *Trypanosoma brucei* (PDB: 1fx2) and GTPase KRas from *Homo sapiens* (PDB: 6god) were identified to have high sequence similarity, i.e., 64.71% and 34.78% for CYCc and RAS1, respectively. In general, a protein sequence with a minimum 30% identity with target protein is adequate to be considered as an appropriate template for accurate homology modeling (Fiser, 2010). Correspondingly, 1fx2 and 6god were selected as templates for building CYCc and RAS1 models. The 3D structures of modeled proteins are shown in **Figures 1A, B**.

Subsequently, the qualities of generated protein models were validated through the Ramachandran plot constructed using PROCHECK module from SAVES Server (https://saves.mbi.ucla.edu/). As can be seen from **Figure 1C**, the predicted 3D structure of CYCc has 95.0%, 12.5%, 0.0%, and 0.0% of residues in the most favored, additional allowed, generously allowed, and disallowed regions, respectively. Similarly, the RAS1 structure has 94.8%, 5.2%, 0.0%, and 0.0% of residues in the most favored, additional allowed, generously allowed, and disallowed regions, respectively (**Figure 1D**). According to the Ramachandran plot, a model structure upholding more than 90% of residues in the most favored region is deemed to be as accurate as like the experimentally solved crystal structure (2-Å resolution) (Sobolev et al., 2020). Interestingly, both the generated models showed ≥90% residues [i.e., CYCc (95.0%) and RAS1 (94.8)] in the most favored region. Besides, both the models did not cover any residue in the disallowed region, which ascertained that the SWISS-MODEL predicted models as reasonable and reliable for further molecular docking studies.

### 3.2 Protein Preparation

Generally, the 3D structures built through homology modeling used to have unfavorable bond lengths, bond angles, torsion angles, and contacts. Therefore, energy minimization was done using UCSF Chimera to normalize local bond length and angle and to remove close contacts in the geometric chain for the predicted proteins. Initially, the refined proteins were energy minimized using AMBER ff14SB and Gasteiger charges for standard and non-standard amino acid residues, respectively. Finally, the minimized protein structures were saved in “.pdb” extension and used for further molecular docking.

The 3D structures of 64 bacterial QSMs were retrieved from SigMoL database (Rajput et al., 2016). Open Babel platform was used to equalize the ligand format to “.sdf” extension, which was collected from different primary databases integrated with SigMoL database. The reformatted ligands were energy minimized with uff force field using Open Babel wizard on PYRX 0.8. The ligands were saved in “.pdbqt” extension for further molecular docking and protein–ligand simulation analyses.

### 3.3 Structure-Based Virtual Screening

As the virtual screening workflow offers selective filtration of ligands (lead drug) that are capable of interacting with protein macromolecules (drug target) with good binding energy, it has been an imperative tool in drug discovery (Maia et al., 2020). In the current study, virtual screening was done for 64 QSMs belonging to five different bacterial QS signaling systems, and the best hits are listed (**Tables 1** and **2**) based on their binding energy toward the selected two seminal drug targets, *viz*., CYCc and RAS1. The obtained binding energies and hydrogen bond interactions of docked ligands with target proteins were summarized in **Tables 1** and **2**.

**Table 1:** List of the 64 screened QSMs with their binding energy and interaction residues against CYCc as predicted through virtual docking.

S. No	SigMolQSSM ID	Ligand name	ID	Energy (kcal mol^−1^)	H-bonding	Signaling system
1.	QSSM 1141	Cyclo(l-Phe-l-4-OH-Pro)	8643197*	−8.2	Asp76, Ser138	DKPs
2.	QSSM 1111	Cyclo(l-Pro-l-Tyr)	106647*	−8.1	Ser138	DKPs
3.	QSSM 1153	Cyclo(l-Phe-*cis*-4-OH-d-Pro)	8976612*	−8.1	Asp76, Ser138	AHLs
4.	QSSM 1157	3-Benzyl-6-isobutylidene-2,5-Piperazinedione	8666841*	−7.9	Asp6, Asp76	DKPs
5.	QSSM 1133	Cyclo(l-Pro-d-Phe)	391657*	−7.6	Ser135	DKPs
6.	QSSM 0615	Cyclo(l-Phe-l-Pro)	90257*	−7.4	Ser138	DKPs
7.	QSSM 0614	*p*-Coumaroyl-HSL	30790745*	−7.3	Lys45, Arg105, Ser135, Ser138	AHL
8.	QSSM 1156	3-Benzylidene-6-isobutyl-2,5-piperazinedione	24725401*	−7.2	Ser135	DKPs
9.	QSSM 0615	Cinnamoyl-HSL	9797494*	−7.2	Arg105, Ser135, Ser138	AHLs
10.	QSSM 0005	*N*-Tetradec-7-enoyl-l-homoserine lactone	28589930*	−7.1	Ser10, Asp49	AHLs
11.	QSSM 1144	Cyclo(Gly-l-Tyr)	19927129*	−7	Lys45, Ser138	DKPs
12.	QSSM 0271	*N*-(3-Oxononanoyl)-l-homoserine lactone	9768814*	−7	Ser10, Thr11	AHLs
13.	QSSM 1132	Cyclo(l-Pro-l-isoLeu)	8166289*	−6.9	Asp76	DKPs
14.	QSSM 1262	2-Heptylquinolin-4(1*H*)-one	164974#	−6.8	Thr74, Ser138	HAQs
15.	QSSM 0619	*N*-Isovaleryl-l-homoserine lactone	29368455*	−6.6	Ser10, Thr11	AHLs
16.	QSSM 0042	*N*-(3-Hydroxydecanoyl)-l-homoserine lactone	9696680	−6.6	Ser10, Thr11, Asp76	AHLs
17.	QSSM 0012	2-Heptyl-3-hydroxy-4-quinolone	2763159#	−6.5	Thr74	HAQs
18.	QSSM 1151	Cyclo(l-Leu-*trans*-4-OH-l-Pro)	10184045*	−6.4	Ala50	DKPs
	QSSM	Farnesol	445070#	−6.4	Asp6, Asp76, Arg105	Fungal QSM
19.	QSSM 1112	Cyclo(l-Pro-l-Leu)	5428292*	−6.4	Thr74	DKPs
20.	QSSM 0739	*N*-(3-Oxotetradec-7-enoyl)-l-homoserine lactone	9057115*	−6.4	Asp76, Ser135	AHLs
21.	QSSM 0891	*N*-Pentanoyl-l-homoserine lactone	9688673*	−6.4	Ser10, Thr11	AHLs
22.	QSSM 0012	N-(3-Oxododecanoyl)-l-homoserine lactone	10221060#	−6.2	Arg100	AHLs
23.	QSSM 0368	*N*-(3-Oxohexadec-9-enoyl)-l-homoserine lactone	29341818*	−6.2	Arg100, Ser135	AHLs
24.	QSSM 1148	Cyclo(d-4-OH-Pro-l-Val)	34017887*	−6.2	Lys45	DKPs
25.	QSSM 0041	*N*-(3-Hydroxydodecanoyl)-l-homoserine lactone (OH-dDHL)	9067166*	−6.2	Thr74, Asp76	AHLs
26.	QSSM 1114	Cyclo(l-Val-l-Leu)	122161*	−6.1	Asp6, Thr11, Asp76	DKPs
27.	QSSM 0234	*N*-Tetradec-2,7-dienoyl-l-homoserine lactone	28589928*	−6.1	Asp76, Ser135	AHLs
28.	QSSM 1194	(2*E*,6*E*)-Farnesoic acid	4439611*	−6.1	Ser135	DSFs
29.	QSSM 0389	*N*-(3-Hydroxytetradec-7-enoyl)-l-homoserine lactone	4445245*	−6.1	Thr74, Asp76, Ser135	AHLs
30.	QSSM 1177	(2*E*,4*E*)-11-Methyl-2,4-dodecadienoic acid	8031542*	−6.1	Ile7, Asn9, Arg105	DSFs
31.	QSSM 0573	3-Oxo-C16-HSL	9683431*	−6.1	Thr74, Ser135	AHLs
32.	QSSM 0046	*N*-(3-Oxooctanoyl) homoserine lactone	127293#	−6	Asp76	AHLs
33.	QSSM 0987	(1*S*,4*S*,5*R*)-7,7-dihydroxy-1-methyl-2,6,8-trioxa-7-boranuidabicyclo[3.3.0]octane-4,5-diol	395434*	−6	Asp6, Thr11, Asp72, Arg105	AI-2
34.	QSSM 0104	*N*-(3-Hydroxyoctanoyl)-l-homoserine lactone	9761556*	−6	Asp76	AHLs
35.	QSSM 0038	*N*-(3-Hydroxybutanoyl)-l-homoserine lactone	115719*	−5.9	Ser138	AHLs
36.	QSSM 0026	3-Hydroxy-*N*-[(3S)-2-oxotetrahydro-3-furanyl]tetradecanamide	9078807*	−5.9	Thr74, Asp76	AHLs
37.	QSSM 0020	*N*-Hexanoyl-l-homoserine lactone	10058590#	−5.8	Ser138	AHLs
38.	QSSM 0013	*N*-Butyryl-l-homoserine lactone	10130163#	−5.8	Asp76	AHLs
39.	QSSM 0048	*N*-Heptanoylhomoserine lactone	443437#	−5.8	Asp76	AHLs
40.	QSSM 0003	*N*-Octanoyl-l-homoserinelactone	6914579#	−5.8	Ser135	AHLs
41.	QSSM 0369	*N*-(Tetrahydro-2-oxo-3-furanyl)-octadecanamide	69757690#	−5.8	Ser135	AHLs
42.	QSSM 1129	Cyclo(Pro-Val)	8997025*	−5.8	Asp6, Arg105	DKPs
43.	QSSM 1129	Cyclo(Pro-Val)	8997025*	−5.8	Asp6, Arg105	DKPs
44.	QSSM 0107	*N*-3-Oxo-tetradecanoyl homoserine lactone	11688418#	−5.7	Arg100, Ser135	AHLs
45.	QSSM 0025	*N*-(3-Hydroxyhexanoyl)-l-homoserine lactone	9754171*	−5.7	Asp76	AHLs
46.	QSSM 1202	Decanoic acid	2863#	−5.6	Asn9, Ser10, Thr11, Arg105	DSFs
47.	QSSM 1191	2-Pentadecenoic acid	4445869*	−5.6	Ser135	DSFs
48.	QSSM 0001	*N*-(beta-Ketocaproyl)-l-homoserine lactone	688505#	−5.6	Ser135	AHLs
49.	QSSM 0764	*N*-(3-Hydroxyhexadecanoyl)-l-homoserine lactone	9741349*	−5.6	Arg100, Ser135	AHLs
50.	QSSM 1175	Decenoic acid	4445851*	−5.5	Asn9, Ser10, Thr11, Arg105	DSFs
51.	QSSM 1190	2-Tetradecenoic acid	4445865*	−5.5	Thr5, Ser138	DSFs
52.	QSSM 1167	11-Methyl-2-dodecenoic acid	9644750*	−5.5	Asp6	DSFs
53.	QSSM 1204	Tridecylic acid	12013*	−5.4	Ser135, Ser138	DSFs
54.	QSSM 0367	*N*-Hexadec-9-enoyl-l-homoserine lactone	28589932*	−5.4	Ser135	AHLs
55.	QSSM 1181	Undecenoic acid	4445855*	−5.4	Lys45, Asp125, Tyr126	DSFs
56.	QSSM 0016	*N*-Hexadecanoyl-homoserine lactone	11609787#	−5.3	Arg100	AHLs
57.	QSSM 1192	Lauric acid	3756*	−5.3	Ser135, Ser138	DSFs
58.	QSSM 0990	4,5-Dihydroxy-2,3-pentanedione	9346083*	−5.3	Asp6, Ser10, Thr11, Asp49, Arg105	AI-2
59.	QSSM 1205	Pentadecanoic acid	13249*	−5.2	Ser134	DSFs
60.	QSSM 1145	Cyclo(Gly-l-Val)	644035*	−5.2	Gln67, Asp118, Asp125	DKPs
61.	QSSM 0289	*N*-(3-Oxononanoyl)-l-homoserine lactone	289	−5.1	Asp6, Ser10, Glu47, Asp49	AHLs
62.	QSSM 1183	2-Tridecenoic acid	4445862*	−5	Ile7, Asn9, Thr11	DSFs
63.	QSSM 1159	2-Dodecenoic acid	4471801*	−5	Thr74, Asp76	DSFs
64.	QSSM 1179	2-Octenoic acid	4445841#	−4.9	Asn9, Thr11, Arg105	DSFs

QSMs, quorum-sensing molecules; DKPs, diketopiperazines; AHLs, acylated homoserine lactones.

**Table 2:** List of the 64 screened QSMs with their binding energy and interaction residues against RAS1 as predicted through virtual docking.

S. no.	SigMol	Ligand name	ID	Energy (kcal mol^−1^)	H-bonding	Signaling system
	QSSM ID					
1.	QSSM 0614	*p*-Coumaroyl-HSL	30790745*	−8.5	Gly16, Lys17, Asp120	AHLs
2.	QSSM 1111	Cyclo(l-Pro-l-Tyr)	106647*	−8.2	Ser18, Asn117	DKPs
3.	QSSM 1141	Cyclo(l-Phe-l-4-OH-Pro)	8643197*	−8.2	Gly14, Glu32, Asp34	DKPs
4.	QSSM 1153	Cyclo(l-Phe-*cis*-4-OH-d-Pro)	8976612*	−8.2	Gly14, Glu32, Asp34	AHLs
5.	QSSM 0615	Cinnamoyl-HSL	9797494*	−8.2	Ser18	AHLs
6.	QSSM 1113	Cyclo(l-Phe-l-Pro)	90257*	−8.1	Asn117	DKPs
7.	QSSM 0234	*N*-Tetradec-2,7-dienoyl-l-homoserine lactone	28589928*	−8	Gly16, Lys17	AHLs
8.	QSSM 1194	(2*E*,6*E*)-Farnesoic acid	4439611*	−8	Val15, Gly14, Lys17	DSFs
9.	QSSM 0389	*N*-(3-Hydroxytetradec-7-enoyl)-l-homoserine lactone	4445245*	−7.9	Val15, Gly16, Lys17, Ser18, Glu32	AHLs
10.	QSSM 0041	*N*-(3-Hydroxydodecanoyl)-l-homoserine lactone (OH-dDHL)	9067166*	−7.9	Gly16, Lys17, Ser18, Val30	AHLs
11.	QSSM 0042	*N*-(3-Hydroxydecanoyl)-l-homoserine lactone	9696680	−7.8	Val15, Gly16, Lys17, Val30, Glu32	AHLs
12.	QSSM 1262	2-Heptylquinolin-4(1*H*)-one	164974#	−7.6	Asn117	HAQs
13.	QSSM 0026	*N*-(3-Hydroxytetradecanoyl)-l-homoserine lactone	9078807*	−7.6	Val15, Gly16, Lys17, Ala19, Glu32	AHLs
14.	QSSM 0104	*N*-(3-Hydroxyoctanoyl)-l-homoserine lactone	9761556*	−7.5	Val15, Gly16, Lys17, Ser18	AHLs
15.	QSSM 1156	3-Benzylidene-6-isobutyl-2,5-piperazinedione	24725401*	−7.4		DKPs
16.	QSSM 0368	*N*-(3-Oxohexadec-9-enoyl)-l-homoserine lactone	29341818*	−7.4	Gly16, Lys17	AHLs
17.	QSSM 1133	Cyclo(l-Pro-d-Phe)	391657*	−7.4	Val30, Lys118	DKPs
18.	QSSM 0764	*N*-(3-Hydroxyhexadecanoyl)-l-homoserine lactone	9741349*	−7.4	Val15, Gly16, Lys17, Glu32	AHLs
19.	QSSM 1151	Cyclo(l-Leu-*trans*-4-OH-l-Pro)	10184045*	−7.3	Asp120, Ser147, Ala148	DKPs
20.	QSSM 0012	*N*-(3-Oxododecanoyl)-l-homoserine lactone	10221060#	−7.3	Gly16, Lys17	AHLs
21.	QSSM 0107	*N*-3-Oxo-tetradecanoyl homoserine lactone	11688418#	−7.3	Gly16, Lys17, Ser18	AHLs
22.	QSSM 0012	2-Heptyl-3-hydroxy-4-quinolone	2763159#	−7.3		HAQs
23.	QSSM 1148	Cyclo(d-4-OH-Pro-l-Val)	34017887*	−7.3	Ser147, Alu148, Lys49	DKPs
24.	QSSM 1157	(*Z*)-3-Benzyl-6-isobutylidene-2,5-piperazinedione	8666841*	−7.3		DKPs
25.	QSSM 0573	3-Oxo-C16-HSL	9683431*	−7.3	Lys17, Ser18	AHLs
26.	QSSM 1144	Cyclo(Gly-l-Tyr)	19927129*	−7.2	Gly14, Lys17, Asp31, Asp34	DKPs
27.	QSSM 0369	*N*-(Tetrahydro-2-oxo-3-furanyl)-octadecanamide	69757690#	−7.2	Gly16, Lys17	AHLs
28.	QSSM 0016	*N*-Hexadecanoyl-homoserine lactone	11609787#	−7.1	Val15, Gly16, Lys17, Ser18	AHLs
29.	QSSM 0046	*N*-(3-Oxooctanoyl) homoserine lactone	127293#	−7.1	Gly16, Lys17	AHLs
30.	QSSM 0271	*N*-(3-Oxononanoyl)-l-homoserine lactone	9768814*	−7.1	Lys17, Ser18, Ala19	AHLs
	QSSM	Farnesol	445070#	−7	Asp120, Ser147, Ala148, Lys149	Fungal QSM
31.	QSSM 1112	Cyclo(l-Pro-l-Leu)	5428292**	−7	Ala148, Lys149, Asp120	DKPs
32.	QSSM 0003	*N*-Octanoyl-l-homoserinelactone	6914579#	−7	Gly16, Lys17, Ser18	AHLs
33.	QSSM 0367	*N*-Hexadec-9-enoyl-l-homoserine lactone	28589932*	−6.8	Ser18, Thr36	AHLs
34.	QSSM 0048	*N*-Heptanoylhomoserine lactone	443437#	−6.8	Gly16, Lys17	AHLs
35.	QSSM 1177	11-Methyl-2,4-dodecadienoic acid	8031542*	−6.8	Val15, Gly16, Lys17, Ser18	DSFs
36.	QSSM 0038	*N*-(3-Hydroxybutanoyl)-l-homoserine lactone	115719*	−6.7	Glu14, Val15, Gly16, Lys17, Ser18	AHLs
37.	QSSM 1205	Pentadecanoic acid	13249*	−6.7	Val15, Gly16, Lys17, Ser18	DSFs
38.	QSSM 0001	*N*-(beta-Ketocaproyl)-l-homoserine lactone	688505#	−6.7	Gly16, Lys17	AHLs
39.	QSSM 1183	2-Tridecenoic acid	4445862*	−6.6	Val15, Gly16, Lys17, Ser18	DSFs
40.	QSSM 1191	2-Pentadecenoic acid	4445869*	−6.6	Val15, Gly16, Lys17, Ser18	DSFs
41.	QSSM 0270	*N*-(3-Oxoheptanoyl)-l-homoserine lactone	9710716*	−6.6	Ser18, Ala19, Asp117	AHLs
42.	QSSM 0619	*N*-Isovaleryl-l-homoserine lactone	29368455*	−6.5	Gly16, Lys17, Ser18	AHLs
43.	QSSM 1190	2-Tetradecenoic acid	4445865*	−6.5	Val15, Gly16, Lys17, Ser18	DSFs
44.	QSSM 1132	Cyclo(l-Pro-l-isoLeu)	8166289*	−6.5	Asn117	DKPs
45.	QSSM 1167	11-Methyl-2-dodecenoic acid	9644750*	−6.4	Glu14, Val15, Gly16, Lys17	DSFs
46.	QSSM 0739	*N*-(3-Oxotetradec-7-enoyl)-l-homoserine lactone	9057115*	−6.3	Gly14, Gly16, Lys17	AHLs
47.	QSSM 0891	*N*-Pentanoyl-l-homoserine lactone	9688673*	−6.3	Gly16, Lys17	AHLs
48.	QSSM 0020	*N*-Hexanoyl-l-homoserine lactone	10058590#	−6.2	Gly16, Lys17, Ser18	AHLs
49.	QSSM 0005	*N*-Tetradec-7-enoyl-l-homoserine lactone	28589930*	−6.2	Ser18	AHLs
50.	QSSM 1181	Undecenoic acid	4445855*	−6.2	Val15, Gly16, Lys17, Ser18	DSFs
51.	QSSM 0025	*N*-(3-Hydroxyhexanoyl)-l-homoserine lactone	9754171*	−6.2	Asn117, Lys118, Ser147, Ala148, Lys149	AHLs
52.	QSSM 1204	Tridecylic acid	12013*	−6.1	Val15, Gly16, Lys17	DSFs
53.	QSSM 1192	Lauric acid	3756*	−6.1	Gly14, Val15, Lys17, Ser18	DSFs
54.	QSSM 1145	Cyclo(Gly-l-Val)	644035*	−6	Gly14, Gly16, Asp34	DKPs
55.	QSSM 1114	Cyclo(l-Val-l-Leu)	122161*	−5.9	Asp31	DKPs
56.	QSSM 0987	(1*S*,4*S*,5*R*)-7,7-Dihydroxy-1-methyl-2,6,8-trioxa-7-boranuidabicyclo[3.3.0]octane-4,5-diol	395434*	−5.9	Ser18, Thr36, Asp34	AI-2
57.	QSSM 1159	2-Dodecenoic acid	4471801*	−5.9	Val15, Gly16, Lys17, Ser18	DSFs
58.	QSSM 1175	Decenoic acid	4445851*	−5.8	Val15, Gly16, Lys17, Ser18	DSFs
59.	QSSM 1129	Cyclo(Pro-Val)	8997025*	−5.8		DKPs
60.	QSSM 0990	4,5-Dihydroxy-2,3-pentanedione	9346083*	−5.8	Gly14, Lys17, Ser18, Asp34, Thr36, Thr59, Gly61	AI-2
61.	QSSM 1202	Decanoic acid	2863#	−5.7	Val15, Gly16, Lys17, Ser18	DSFs
62.	QSSM 0289	*N*-(3-Oxononanoyl)-l-homoserine lactone	289	−5.7	Asp120, Ser147, Ala148, Lys149	AHLs
63.	QSSM 0013	*N*-Butyryl-l-homoserine lactone	10130163#	−5.5	Gly16, Lys17	AHLs
64.	QSSM 1179	2-Octenoic acid	4445841#	−5.4	Val15, Gly16, Lys17, Ser18	DSFs

QSMs, quorum-sensing molecules; DKPs, diketopiperazines; AHLs, acylated homoserine lactones.

In this study, a well-known antihyphal QSM of *C. albicans*, *viz*., farnesol, was used as the positive control. It showed a binding affinity of −6.4 and −7.0 kcal mol^−1^ toward CYCc and RAS1, respectively. Two filtration processes were done for 64 QSMs to select one best hit against each of the two target proteins. Initially, the top three lead molecules were selected. However, on comparing the binding affinity and binding profile with reference molecule farnesol, the best hit was identified.

#### 3.3.1 Quorum-Sensing Molecules’ Interaction Mode Against CYCc

As shown in **Table 1**, the binding affinity of docked QSMs against the target protein CYCc ranged from −4.9 to −8.2 kcal mol^−1^. The maximum binding affinity of −8.2 kcal mol^−1^ was displayed by a QSM named cyclo(l-Phe-l-4-OH-Pro), which belongs to DKP signaling system. The initial selection was based on the binding affinity toward the target protein in which out of 31 AHLs, 15 DKPs, 14 DSFs, 2 HAQs and 2 AI-2, 7 AHLs, 9 DKPs, and 2 HAQs were considered for selecting the top three hits, as they exhibited higher binding affinity than the farnesol. On the other hand, the QSMs belonging to AI-2 and DSFs displayed considerably lower binding energy than the positive control farnesol, and hence, these were excluded for the selection of the top three hits.

**Figure 2:**
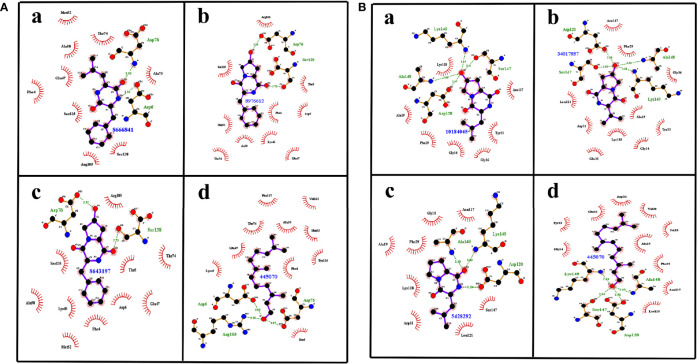
**(A)** The two-dimensional interaction image showcasing the binding pattern of selected top three ligands such as QSSM 1157 (a), QSSM 1141 (b), and QSSM 1153 (c) and the positive control farnesol (d) against CYCc. **(B)** The two-dimensional interaction pattern of selected top three ligands such as QSSM 1151 (a), QSSM 1148 (b), and QSSM 1112 (c) and the positive control farnesol (d) against RAS1.

Apart from the binding energy, the binding profile of QSMs that closely resembles farnesol’s binding profile was considered as the chief criterion to select the top three lead molecules. Based on this, QSMs 3-benzyl-6-(2-methylpropylidene)-2,5-piperazinedione (QSSM 1157), cyclo(l-Phe-l-4-OH-Pro) (QSSM 1141), and cyclo(l-Phe-*cis*-4-OH-d-Pro) (QSSM 1153) were selected as the top three hits, which showed the binding affinity of −8.2, −8.1, and −7.9 kcal mol^−1^, respectively (**Figure 2A**).

The positive control (farnesol) exhibited a strong binding affinity toward CYCc with the docking score of −6.4 kcal mol^−1^. Farnesol interacted with CYCc by forming three hydrogen bonds with amino acid residues of Asp6, Asp76, and Arg105 at 3.05, 3.07, and 2.80 distances, respectively. Furthermore, it showcased 10 hydrophobic interactions with Phe4, Thr 5, Lys45, Glu47, Ala50, Met52, Thr74, Tyr126, Phe127, and Val131 (**Figure 2Ad**).

First, the ligand QSSM 1157 isolated from bacterium *Streptomyces albulus* has shown two hydrogen bonds with the residues of Asp6 and Asp76 at 3.02 and 3.12 distances, respectively. It also showed nine hydrophobic bonds with the residues of Phe4, Glu47, Ala50, Met52, Thr74, Ala75, Arg105, Ser135, and Ser138. Notably, *S. albulus* has already been reported to be a good producer of the fungicide cycloheximide (**Figure 2Aa**) (Yin et al., 2014). The antifungal effect of *S. albulus* synthesized dipeptides; i.e., ε-polylysine was demonstrated against *C. albicans* (Shima et al., 1984). In addition, the antibiofilm activity of a metabolite produced by other *Streptomyces* species, i.e., *Streptomyces chrestomyceticus*, has been reported against *C. albicans* (Srivastava and Dubey, 2016). Taken together, the present docking result with other existing investigations suggests the plausible existence of intercommunication cross-talk between *Streptomyces* species and *C. albicans*, which in a deeper sense certainly requires further investigation.

The second top hit molecule QSSM 1141 belonging to DKP signaling system has been documented to be produced by Gram-negative proteobacterium *Ruegeria* spp. It formed two hydrogen bonds with Asp76 and Ser138 at 2.92 and 2.75 distances, respectively. It also formed 10 hydrophobic interactions with the amino acid residues of Phe4, Thr 5, Asp6, Lys45, Glu47, Ala50, Met52, Thr74, Arg105, and Ser135 (**Figure 2Ab**).

QSSM 1153, also belonging to DKP signaling system and isolated from soil-dwelling Gram-positive bacterium *Saccharothrix espanaensis*, has the required structure to interact through two hydrogen bonds with Asp76 and Ser138 at 2.96 and 2.73 distances, respectively. Similar to QSSM 1141, it can also form 10 hydrophobic bonds with the amino acid residues of Phe4, Thr 5, Asp6, Lys45, Glu47, Ala50, Met52, Thr74, Arg105, and Ser135. It is noteworthy to mention here that a new class of antibiotics, namely, saccharomicins produced by *S. espanaensis*, has already been reported to have potent antimicrobial efficacy against certain pathogenic bacteria and yeast (**Figure 2Ac**) (Singh et al., 2000).

Based on the farnesol pattern of hydrogen bond formation and non-hydrophobic interactions with the target proteins CYCc and RAS1, the top three QSMs were examined, and one best ligand molecule was selected. Accordingly, QSSM 1157 was selected as the best ligand molecule among the three DKPs. It can establish two hydrogen bonds (at Asp6 and Asp76) and five non-hydrophobic bonds (at Phe4, Glu47, Ala50, Met52, and Thr74) at the same position as like farnesol with a greater binding affinity score. Hence, this ligand molecule was taken forward for MD analysis to understand its interaction with CYCc protein.

#### 3.3.2 Quorum-Sensing Molecules’ Interaction Mode Against RAS1

As tabulated (**Table 2**), the 64 docked QS ligand molecules displayed a range of binding affinity with a maximum of −8.5 kcal mol^−1^ and a minimum of −5.4 kcal mol^−1^ against the modeled crystal structure of RAS1 protein. In particular, a QSM named *p*-coumaroyl-HSL belonging to the AHL system demonstrated the highest binding affinity (−8.5 kcal mol^−1^). In parallel, the reference molecule (farnesol) exhibited strong binding interactions toward RAS1 with a binding energy of −7 kcal mol^−1^ (**Figure 2B**).

For the identification of the top three hits against RAS1, the same selection criterion used for the CYC1 target protein was employed. Accordingly, the QSMs belonging to DKP (9), AHL (18), and HAQ (2) QS signaling systems were selected and considered for the next level of screening, as they displayed better binding energy than the reference molecule. On the other hand, the QSMs of AI-2 and DSFs showcased marginal binding energy than the biomolecules of other QS systems and reference molecules, and hence they were excluded from further consideration.

From the analysis, it was evident that QSMs cyclo(l-Pro-l-Leu) (QSSM 1112), cyclo(l-Leu-*trans*-4-OH-l-Pro) (QSSM 1151), and cyclo(d-4-OH-Pro-l-Val) (QSSM 1148) with binding energies of −7, −7.3, and −7.3 kcal mol^−1^ occupy the top three hit positions, as they showcased accurate binding poses in comparison with farnesol (**Figure 2Bd**). The positive control (farnesol) interacts with the RAS1 protein by establishing four hydrogen bonds with the amino acid residues of Asp120, Ser147, Ala148, and Lys149 at the distances of 2.91, 2.86, 2.97, and 2.89, respectively.

QSSM 1112 isolated from soil-dwelling Gram-positive bacterium *S. espanaensis* could establish three hydrogen bonds with the amino acid residues of Asp120, Ser147, and Ala148 at the distances of 2.97, 3.06 and 3.19, respectively. In addition, it formed six hydrophobic bonds with Gly14, Gly16, Ala19, Phe29, Tyr33, Asn117, and Lys118 (**Figure 2Ba**).

The QS molecule QSSM 1151 has been identified from various bacterial sources, namely, *Pseudomonas putida*, *Burkholderia cepacia*, *Shewanella baltica*, and *Streptomyces* spp. It stabilized the interaction with RAS1 through three hydrogen bonds with Ala148, Lys149, and Asp120 (at binding distances of 3.10, 3.10, and 3.05, respectively) and eight hydrophobic bonds with amino acid residues of Gly16, Ala19, Phe29, Asp31, Asn117, Lys118, Leu121, and Ser147 (**Figure 2Bb**). A study conducted by Li et al. (2007) revealed the antifungal efficacy of a novel compound from *B. cepacia*. Further, *B. cepacia* has been reported as a source of production of antibiotics such as pyrrolnitrin, altericidins, and cepacin. Furthermore, another study signified the antifungal efficacy of extract from *Streptomyces* spp. against *C. albicans* (Human et al., 2016). Notably, the antagonistic mechanism of *B. cepacia* through its signaling molecules, i.e., BDSF, toward *C. albicans* has already been well investigated by Boon et al. (2008), since the QSM of *B. cepacia* showed a better binding interaction with RAS1, which further signifies the possible interspecies communication-mediated regulation of *C. albicans* virulence dimorphism by *B. cepacia* QSM.

Similar to QSSM 1141, QSSM 1148 is synthesized by the Gram-negative proteobacterium *Ruegeria* spp. It interacts with RAS1 by forming three hydrogen bonds with Ser147, Alu148, and Lys49 and 10 hydrophobic bonds with Gly14, Gly16, Ala19, Phe29, Glu32, Tyr33, Asn117, Lys118, and Leu121 (**Figure 2Bc**).

Out of the top three hits, the best one QSM against RAS1 was selected based on the same interaction criteria followed for CYCc. As a result, the QS ligand molecule QSSM 1112 was selected as the best ligand, as it established four strong hydrogen Q18 bonds (at Asp120, Ser147, Ala148 and Lys 149) and six non-hydrophilic bonds (Gly14, Gly16, Ala19, Phe29, Tyr33 and Asn117 ) with RAS1 and taken forward to investigate the QSSM 1112-RAS1 complex stability.

**Figure 3:**
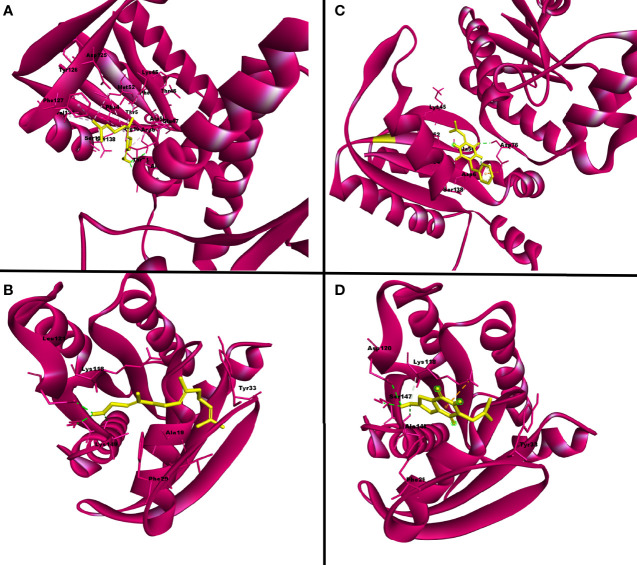
The three-dimensional binding pattern of top hits QSSM 1157 **(C)** QSSM 1112 **(D)** and positive control farnesol **(A, B)** against CYCc and RAS1.

Overall, out of 64 QSMs belonging to five different QS signaling systems used in the virtual docking analysis, two QSMs, *viz*., QSSM 1157 and QSSM 1112, belonging to DKPs were selected as the best hits against CYCc and RAS1, respectively. The 3D interaction of QSSM 1157 and QSSM 1112 against CYCc and RAS1 is shown in **Figure 3**. Moreover, the data of virtual screening obviously demonstrated the phenomenal binding efficacy of QSMs from DKP QS system than other docked signaling systems. In contrast, a couple of earlier studies have reported the inhibitory efficacy of AHLs as well as DSFs toward *C. albicans* hyphal dimorphism in a dose-dependent manner (Zhang et al., 2011; Grainha et al., 2020).

Despite having better binding energy, AHL could not occupy the top hit list owing to its weak binding profile when compared with farnesol. On the other hand, the well-reported structural and functional farnesol analogs of DSFs have displayed a very weak binding energy among the used signaling systems. The weak binding profile of DSFs toward target proteins (CYCc and RAS1) could be because of a distinct antihyphal mode of action different from that of the action mechanism of farnesol. In line with this, a previous finding has also demonstrated that, unlike AHLs and farnesol, the antihyphal mechanism of BDSFs (a *B. cenocepacia* QSM) follows a different mode of action (Hall et al., 2011).

The data of the current study unveiled the excellent binding mode of DKPs over essential hyphal proteins. DKPs are the simplest cyclic dipeptides that have been utilized for myriads of pharmaceutical propensities over the past few decades (de Carvalho and Abraham, 2012). The drug likeliness properties of DKPs attracted the attention of researchers to use them for a wide range of biological activities including antitumor, antiviral, antifungal, antibacterial, and antihyperglycemic efficacies (Rhee, 2004). Besides, the QS intervening efficacy of DKPs by acting as a QS signaling molecule during bacterial cross-talk has been reviewed critically by Bellezza et al. (2014).

### 3.4 Molecular Dynamics and Simulation

In order to understand the stability and interatomic motions of CYCc-QSSM 1157 and RAS1-QSSM 1112 complexes, MD simulation analysis was performed using GROMACS, which would offer essential information regarding the physical movement of atoms and molecules in a biophysical system using computational approaches (Barnes and Williams, 1996). Therefore, the predicted protein–ligand complexes, i.e., CYCc-QSSM 1157 and RAS1-QSSM 1112 complexes, were subjected to 50-ns MD simulation. The subsequent MD run trajectories were examined for protein and ligand RMSDs, protein RMSF, and hydrogen bond interactions.

#### 3.4.1 Root Mean Square Deviation and Root Mean Square Fluctuation Analyses

**Figure 4:**
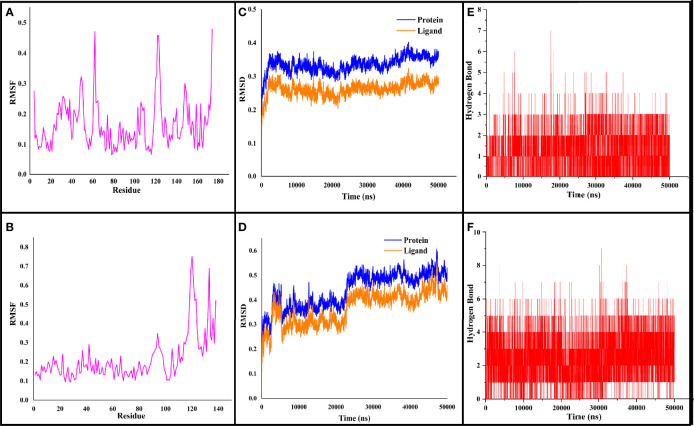
The RMSF, RMSD, and hydrogen bond values plotted for CYCc-QSSM 1157 **(A–C)** and RAS1-QSSM 1112 complexes **(D–F)** during 50-ns MD simulation. RMSF, root mean square fluctuation; RMSD, root mean square deviation; MD, molecular dynamics.

The stabilized interaction of protein–ligand complexes during MD simulation was evaluated by estimating their respective RMSD. Generally, the higher RMSD value signifies the instability of inspected molecules due to the high level of conformational changes within the complex. Additionally, the ligand molecule with a higher RMSD value against its respective protein–ligand complexes would suggest the insufficient binding of the ligand to the target protein (Opo et al., 2021). Thereby, RMSD analysis would reveal how often a protein–ligand interaction is integrated and stabilized. As shown in **Figures 4C, D**, the protein maintained a steady RMSD throughout the simulation run. The CYCc-QSSM 1157 and RAS1-QSSM 1112 protein complexes begin to stabilize after 5 and 25 ns of MD simulation run, respectively. The average protein backbone RMSD for the CYCc-QSSM 1157 and RAS1-QSSM 1112 protein complexes was determined to be 0.42 and 0.31 Å, respectively. The average ligand RMSD for the CYCc-QSSM 1157 and RAS1-QSSM 1112 protein complexes was determined to be 0.35 and 0.27 Å, respectively. The plot in **Figures 4C, D** indicates that the ligand RMSD is not substantially greater than the protein RMSD, which signifies that the ligand does not diffuse away from the original binding site. The RMSD of MD simulation and molecular docking revealed that the attained complexes CYCc-QSSM 1157 and RAS1-QSSM 1112l are stable in their conformations.

To gain more insights into the stability of modeled proteins, the RMSF was estimated during the MD simulation. The RMSF of the Cα-atoms demonstrated the residual flexibility of each protein. Further, the results also showed the amino acid oscillations in catalytic and non-catalytic sites. For CYCc-QSSM 1157 and RAS1-QSSM 1112, the average protein RMSF was in the range of 0.1–0.73 and 0.06–0.47 Å, respectively (**Figures 4A, B**). Further, negligible fluctuations were observed in disallowed and looping regions of CYCc and RAS1 proteins, which obviously signifies the stability of both predicted proteins throughout the simulation reactions.

#### 3.4.2 H-Bond Interaction Analysis

As the effective binding of ligand to the target protein is mainly attributed to H-bond formation, the QSSM 1157 and QSSM 1112 were monitored for H-bond formation toward CYCc and RAS1 proteins throughout the simulation run. To identify the system stability during the simulation procedure, the number of hydrogen bonds formed vs. simulation duration was calculated. The hydrogen bonds stability was evaluated by means of 50-ns molecular simulation (**Figures 4E, F**). The maximum number of hydrogen bonds formed for CYCc-QSSM 1157 and RAS1-QSSM 1112 was 9 and 7, respectively, indicating the greater affinity of ligand attachment to the protein binding pockets. The MD calculated hydrogen bond formation was in agreement with the result of molecular docking analysis. The number of hydrogen bonds formed by the QSSM 1157 and QSSM 1112 over CYCc and RAS1 throughout the simulation showed enough ligand binding strength to the proteins. Furthermore, the hydrogen bond ensures greater affinity and stability of ligand to the proteins.

### 3.5 Pharmacokinetic Prediction Using SwissADME Server

In the *de novo* drug development process, most of the drugs have failed in the final clinical phase due to their inappropriate pharmacokinetics and toxicity profile. To overcome this issue, pharmacokinetic and ADME prediction of the new drug candidate has to be conducted at the initial stages (Lee et al., 2003). To attain a desirable *in vivo* response, there should be an equilibrium between pharmacokinetic and pharmacodynamic qualities (Tuntland et al., 2014). Presently, *in silico*-based ADME screening is being utilized for the identification of the most promising compounds devoid of drug attrition.

In the present study, numerous parameters such as cell permeability, drug solubility, partition coefficient, gastrointestinal (GI) absorption, polar surface area (PSA), and drug homology were evaluated through virtual screening approaches provided by SwissADME interface. The results obtained in SwissADME are listed in **Table 3**, **Supplementary Table 1**, which contain ADMET, pharmacokinetics, drug likeliness, and medical chemistry of predicted ligands QSSM 1157 and QSSM 1112.

**Table 3:** Pharmacokinetics, drug likeness, and medicinal chemistry properties of QSSM 1157 and QSSM 1112 as predicted through SwissADME.

Parameters	QSSM 1112	QSSM 1157
**Pharmacokinetics**		
GI absorption	High	Low
BBB permeation	No	No
P-gp substrate	No	No
CYP1A2 inhibitor	No	Yes
CYP2C19 inhibitor	No	No
CYP2C9 inhibitor	No	No
CYP2D6 inhibitor	No	No
CYP3A4 inhibitor	No	No
**Drug likeness**		
Lipinski	Yes; 0 violation	Yes; 0 violation
Ghose	Yes; 0 violation	Yes; 0 violation
Veber	Yes	Yes
Egan	Yes	Yes
Muegge	Yes	Yes
Bioavailability Score	0.55	0.55
**Medicinal chemistry**		
PAINS	0 alert	0 alert
Brenk	0 alert	1 alert: michael_acceptor_1
Leadlikeness	Yes; 0 violation	Yes; 0 violation

GI, gastrointestinal; BBB, blood–brain barrier; P-gp, permeability glycoprotein; PAINS, Pan-Assay Interference compounds.

#### 3.5.1 Drug Likeliness Prediction

Drug likeness of a ligand molecule is predicted by comparing the structure or other merit parameters with known drugs. In SwissADME, the drugability of the ligands was evaluated by comparing various properties with pre-developed drug likeliness modules such as Lipinski’s rule of five, Ghose’s rule, Veber’s rule, Egan’s rule, and Muegge’s rule. Each of the mentioned modules contains different parameters that estimate ligand drug likeliness. Molecular weight, hydrogen donors, hydrogen acceptors, rotatable bonds, and PSA are various ligand properties that have been taken into account to evaluate a ligand drug’s transport characteristics.

As can be seen in **Table 3**, the structures of QSSM 1157 and QSSM 1112 possess a better drug likeliness without any deviation in Lipinski’s rule of five, Ghose’s rule, Veber’s rule, Egan’s rule, and Muegge’s rule as their values fall in the desirable range. To be precise, all the parameters, *viz*., molecular weight, rotatable bonds, number of hydrogen acceptors, number of hydrogen donors, PSA, molar refractivity, log P, and clogP, have fallen within the acceptable range. Furthermore, the QSSM 1157 and QSSM 1112 showed better lipophilicity (XLOGP3 between −0.7 and +5.0) and good water solubility scores. This result confirms that the chemical structure and orientation of both ligands will not cause any irrelevant metabolic interactions during drug formulation.

#### 3.5.2 Pharmacokinetics Prediction

The SwissADME pharmacokinetics evaluation revealed that both the ligands have no blood–brain barrier (BBB) permeation and no substrate of permeability glycoprotein (P-gp) (**Table 3**). Furthermore, the QSSM 1157 and QSSM 1112 showed high and low GI absorption, respectively.

The cytochrome P450 (CYP) is a hemeprotein that is responsible for the metabolism of drugs at various stages. Usually, CYP will have exhibited as five major isomers such as CYP1A2, CYP3A4, CYP2C9, CYP2C19, and CYP2D6. Inhibition of CYP pathway may lead to unwanted toxic effects due to reduced drug metabolism (Lynch and Price, 2007). Hence, the property of QSSM 1157 and QSSM 1112 to inhibit CYP was evaluated. The CYP interaction results showed that the QSSM 1112 is not a CYP inhibitor, whereas QSSM 1157 is an inhibitor of one of the isomers of CYP1A2. The obtained result clearly evidences that both QSSM 1157 and QSSM 1112 have a lesser possibility to interfere with CYP, which guarantees the drug selectivity and bioavailability.

The Pan-Assay Interference compounds (PAINS) and Brenk technique were used to identify potentially toxic and unstable fragments present in the intact structure. In many biochemical and pharmacological trials, the PAINS evaluate the misleading hit compounds showing false-positive biological yields (Baell and Nissink, 2018). As expected, both QSSM 1157 and QSSM 1112 had “zero alerts” in PAINS prediction.

Although the QSSM 1112 does not have any violation in the medicinal chemistry profile, the Brenk list predicted QSSM 1157 as a Michael acceptor, indicating the possibility for a hazardous interaction of its fragments with other biological molecules. Since the QSSM 1157 showed inadequate toxicity in the Brenks–Michael acceptor alert, it is a necessity to evaluate the complete toxicity profile of the screened ligands. Hence, it is necessary to evaluate the toxicity profile of the screened ligands.

### 3.6 Toxicity Prediction *via* ProTox-II Server

The prediction of chemical toxicity is a crucial step in the drug development process. Computational-based toxicity prediction plays a significant role in fairly reducing the time and the number of animals to be used in *in vivo* studies for toxicity prediction (Raies and Bajic, 2016). ProTox-II uses 33 various models for predicting molecular similarity, fragment propensities, and machine learning to predict various toxicity endpoints including acute toxicity, hepatotoxicity, cytotoxicity, carcinogenicity, mutagenicity, and immunotoxicity.

**Figure 5:**
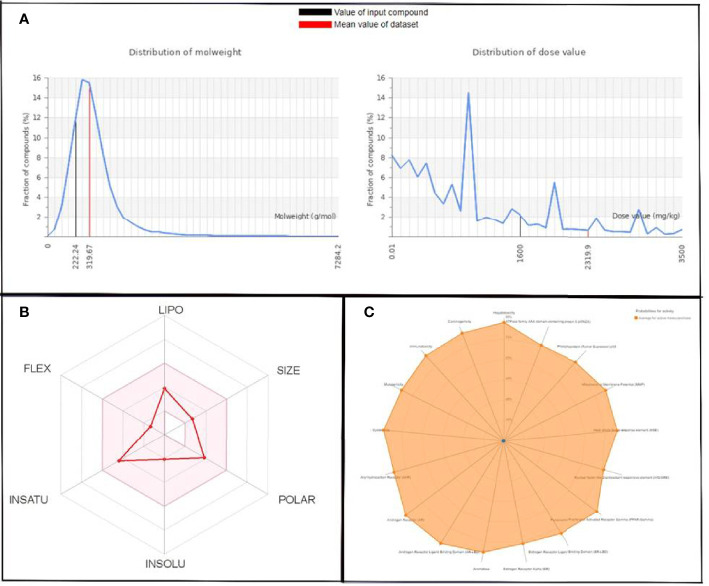
**(A)** Graphical representation of molecular weight and dose value distribution of QSSM 1157. **(B)** Swiss-ADME generated bioavailability radar chart for QSSM 1157. Pink area indicates the oral bioavailability of QSSM 1157. **(C)** Radar chart showcasing various toxicity profiles of QSSM 1157 predicted through ProTox-II.

**Figure 6:**
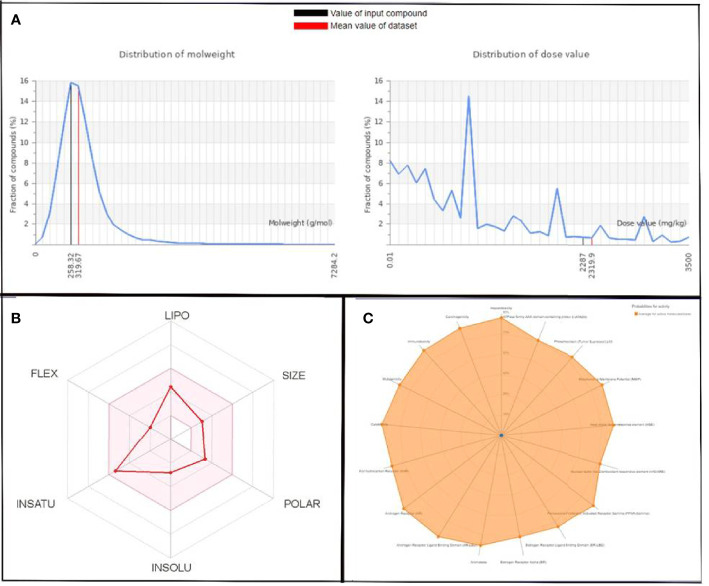
**(A)** Graphical representation of molecular weight and dose value distribution of QSSM 1112. **(B)** Swiss-ADME generated bioavailability radar chart for QSSM 1112. Pink area indicates the oral bioavailability of QSSM 1112. **(C)** Radar chart demonstrating various predicted toxicity profiles of QSSM 1112 through ProTox-II.

The molecular weight distribution profile of both ligands was estimated by the ProTox-II server, and it was in the range of 258.32 to 319.67 for QSSM 1157 and 222.24 to 319.67 for QSSM 1112 (**Figures 5A**, **6A**).

The toxicity was estimated by recognizing the ligand fragments and structure. The various toxicity profiles of both ligands estimated through ProTox-II server are given in **Tables 4**, **5** and **Figures 5**, **6**. The toxicity radar chart in **Figures 5C**, **6C** was intended to quickly illustrate the confidence of positive toxicity results compared with the average of its class. The LD50 values of QSSM 1112 and QSSM 1157 were predicted to be 1,600 and 2,287 mg/kg, respectively. The distribution of dose values for QSSM 1112 and QSSM 1157 with its mean dose value is represented in **Figures 5A**, **6A**, respectively.

**Table 4:** Different toxicity profile of QSSM 1157 predicted through ProTox-II server.

Classification	Target	Prediction	Probability
Organ toxicity	Hepatotoxicity	Inactive	0.71
Toxicity endpoints	Carcinogenicity	Inactive	0.69
Toxicity endpoints	Immunotoxicity	Inactive	0.98
Toxicity endpoints	Mutagenicity	Inactive	0.70
Toxicity endpoints	Cytotoxicity	Inactive	0.67
Tox21-Nuclear receptor signaling pathways	Aryl hydrocarbon receptor (AhR)	Inactive	0.81
Tox21-Nuclear receptor signaling pathways	Androgen receptor (AR)	Inactive	0.96
Tox21-Nuclear receptor signaling pathways	Androgen receptor ligand binding domain (AR-LBD)	Inactive	0.99
Tox21-Nuclear receptor signaling pathways	Aromatase	Inactive	0.95
Tox21-Nuclear receptor signaling pathways	Estrogen receptor alpha (ER)	Inactive	0.93
Tox21-Nuclear receptor signaling pathways	Estrogen receptor ligand binding domain (ER-LBD)	Inactive	0.96
Tox21-Nuclear receptor signaling pathways	Peroxisome proliferator-activated receptor gamma (PPAR-Gamma)	Inactive	0.97
Tox21-Stress response pathways	Nuclear factor (erythroid-derived 2)-like 2/antioxidant responsive element (nrf2/ARE)	Inactive	0.97
Tox21-Stress response pathways	Heat shock factor response element (HSE)	Inactive	0.97
Tox21-Stress response pathways	Mitochondrial Membrane Potential (MMP)	Inactive	0.89
Tox21-Stress response pathways	Phosphoprotein (tumor suppressor) p53	Inactive	0.87
Tox21-Stress response pathways	ATPase family AAA domain-containing protein 5 (ATAD5)	Inactive	0.96

**Table 5:** Different toxicity profile of QSSM 1112 predicted through ProTox-II server.

Classification	Target	Prediction	Probability
Organ toxicity	Hepatotoxicity	Inactive	0.68
Toxicity endpoints	Carcinogenicity	Inactive	0.63
Toxicity endpoints	Immunotoxicity	Inactive	0.97
Toxicity endpoints	Mutagenicity	Inactive	0.75
Toxicity endpoints	Cytotoxicity	Inactive	0.69
Tox21-Nuclear receptor signaling pathways	Aryl hydrocarbon receptor (AhR)	Inactive	0.87
Tox21-Nuclear receptor signaling pathways	Androgen receptor (AR)	Inactive	0.95
Tox21-Nuclear receptor signaling pathways	Androgen receptor ligand binding domain (AR-LBD)	Inactive	0.95
Tox21-Nuclear receptor signaling pathways	Aromatase	Inactive	0.88
Tox21-Nuclear receptor signaling pathways	Estrogen receptor alpha (ER)	Inactive	0.94
Tox21-Nuclear receptor signaling pathways	Estrogen receptor ligand binding domain (ER-LBD)	Inactive	0.96
Tox21-Nuclear receptor signaling pathways	Peroxisome proliferator-activated receptor gamma (PPAR-Gamma)	Inactive	0.92
Tox21-Stress response pathways	Nuclear factor (erythroid-derived 2)-like 2/antioxidant responsive element (nrf2/ARE)	Inactive	0.86
Tox21-Stress response pathways	Heat shock factor response element (HSE)	Inactive	0.86
Tox21-Stress response pathways	Mitochondrial membrane potential (MMP)	Inactive	0.84
Tox21-Stress response pathways	Phosphoprotein (tumor suppressor) p53	Inactive	0.75
Tox21-Stress response pathways	ATPase family AAA domain-containing protein 5 (ATAD5)	Inactive	0.92

The radar chart shows the bioavailability of the selected hit ligands. The pink area of the radar indicates the ideal area for properties such as flexibility (FLEX), insolubility (INSOLU), unsaturation (INSATU), lipophilicity (LIPO), polarity (POLAR), and SIZE. Both the selected ligands match with the recommended size of 500 g mol^−1^ as indicated by Lipinski for successful medication candidates. The POLAR was assessed for the selected ligands using their total area of polarity (TPSA). The TPSA value of a polar molecule should be 20 and 130 Å. The TPSA of QSSM 1157 and 1112 falls within the allowed range. Through INSOLU of the target ligands depicted with their respective ESOL and ESOL Class, it was found that QSSM 1157 and QSSM 1112 displayed high and moderate water-soluble properties, respectively.

The carbon fraction Sp3 (CSP3) and the rotatable bond count should not be greater than 9 and should be within the range of 0.5–1 (CSP3). It has been used to assess the INSATU and FLEX of the identified hit ligands. Both the selected hits QSSM 1157 (CSP3:0.33) and QSSM 1112 (CSP3: 0.45) showed CSP3 values fairly near csp3 range. In general, the numbers of rotatable bonds should not exceed nine; data showed that QSSM 1157 and QSSM 1112 possess 3 and 2 rotatable bonds, respectively. XLogP3 and ESOL (Log S) with the suggested range of − 0.7 to + 5.0 and 0 to 6, respectively, were used to access the LIPO and INSOLU profile of the selected ligands. Interestingly, both the selected ligands fall within the recommended range. Taken together, the data of pharmacokinetic and pharmacodynamic analyses of the selected hit molecules underline their highest plausibility for drug development.

Both the active agents were analyzed for various toxicity profiles and are listed in **Tables 4**, **5**. QSSM 1157 showed mild toxicity possibility rates in hepatotoxicity, carcinogenicity, and cytotoxicity profiles, whereas QSSM 1112 demonstrated mild toxicity rates in carcinogenicity and cytotoxicity profiles.

As listed in **Tables 4**, **5**, both ligands showed no toxicity in Tox21 receptor signaling pathway profiles. The overall toxicity class was predicted to be 4 out of 6 for QSSM 1112 and 5 out of 6 for QSSM 1157, which indicates the lesser toxicity of ligands. Overall, the analyses unambiguously predict that the identified QSM-based hyphal inhibitors would be safe for biological use.

### 3.7 *In Vitro* Antihyphal Assay Revealed Dose-Dependent Inhibitory Potency of QSSM 1112 Against *Candida albicans* Dimorphism

At the end of the virtual screening, the obtained result clearly showed that QSSM 1157 and QSSM 1112 have a similar kind of interaction like farnesol against CYCc and RAS1, respectively. Among the two predicted hits, biomolecule QSSM 1112 (active against RAS1) has been well studied and documented by our research group for its remarkable quorum quelling, antibiofilm, and antivirulence efficacies toward various multidrug-resistant pathogens, *viz*., *S. mutans* (Gowrishankar et al., 2014), *S. aureus* (Gowrishankar et al., 2016a), *Listeria monocytogenes* (Gowrishankar et al., 2016b), *Staphylococcus epidermidis*, and *Serratia marcescens* (Gowrishankar and Pandian, 2017; Gowrishankar et al., 2019). This information led us to be more curious to validate the *in vitro* efficiency of QSSM 1112 in inhibiting yeast-to-hyphal transition due to its in-house availability. Quite a few earlier studies on QSSM 1112 have already been reported for its synthesis (from *Achromobacter xylosoxidans* and *Streptomyces* sp.) and exhibition of antagonistic activity against aflatoxins of *Aspergillus parasiticus* and rice blast fungus *Pyricularia oryzae* (Yan et al., 2004; Hall et al., 2011). Except for the anticandidal effect of QSSM 1112 (Rhee, 2004), so far, none of the studies has been focused on its inhibitory potency toward *C. albicans* virulence dimorphism. Hence, in order to accomplish our curiosity in validating the *in silico* docking result, *in vitro* antihyphal assay was performed using QSSM 1112, with farnesol as the positive control.

**Figure 7:**
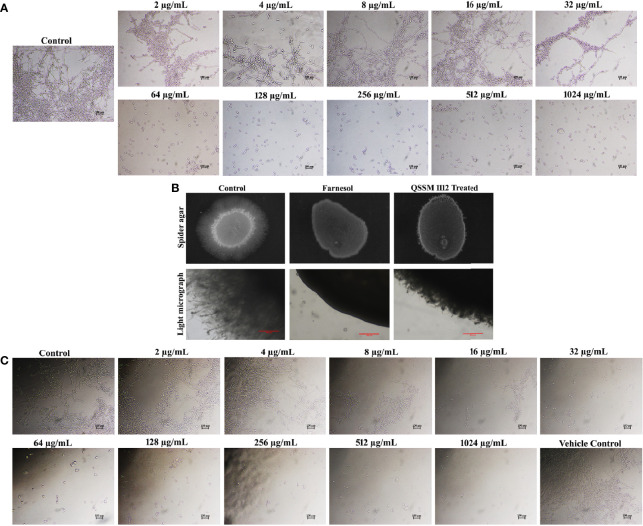
**(A)** Inhibitory efficacy of QSSM 1112 (0–1,024 µg/ml) on yeast-to-hyphal transition of *Candida albicans* in liquid spider media. After 24-h manifestation with QSSM 1112, *C. albicans* cells were photographed under a light microscope at ×400 magnification. Micrograph of the control group portrays very dense and lengthy filamentous cells with few yeast cells; the QSSM 1112-treated groups display more number of evenly distributed yeast cells even from the lowest concentration (64 µg/ml). **(B)** Conformation of yeast-to-hyphal inhibition by QSSM 1112 using solid spider medium. The fungal colonies were imaged after 7 days of incubation at 37°C. The light micrograph and agar plate images clearly portray the remarkable reduction of hyphal protrusion in QSSM 1112-treated samples compared with untreated control (which bares deep and densely elongated hyphal network). **(C)** The impact of positive control farnesol (0–1,024 µg/ml) on *C. albicans* yeast-to-hyphal transition. The last panel in the figure showcases the effect of solvent (methanol) on yeast-to-hyphal transition.

For this, *C. albicans* cells were cultured with and without the active agent (QSSM 1112) in liquid spider medium supplemented with 10% of hyphal inducer (FBS). As expected, the result of the liquid antihyphal assay signified that QSSM 1112 was potent enough to inhibit the yeast-to-hyphal transition in a concentration-dependent manner (**Figure 7A**). Of the various concentrations (0–1,024 µg/ml) used, it was found that QSSM 1112 successfully thwarts the hyphal formation from the maximum (1,024 µg/ml) to minimum (64 µg/ml) concentrations. Therefore, the lowest concentration that brings a substantial hyphal inhibitory condition was considered to be the minimal HIC. Consequently, a concentration of 64 µg/ml was considered as HIC of QSSM 1112, as it demonstrated a phenomenal antihyphal efficacy in a similar fashion to higher concentration (1,024 µg/ml) as well. As depicted in **Figure 7A**, the cells devoid of active compound (untreated control) have displayed a highly dense criss-cross architecture of hyphal cell population along with few yeast cells. On the other hand, the cells exposed with QSSM 1112 have showcased only yeast cell population even under strong hyphae-induced conditions, which clearly proves that the existence of QSSM 1112 disarmed the *C. albicans* cells to undergo hyphae induction. Moreover, the consistency of QSSM 1112 in expressing the sustained hyphal inhibitory potency even in the presence of serum would make the QSSM 1112 an interesting drug candidate in the treatment against invasive *C. albicans* infection.

Farnesol was used as the positive control in all the *in vitro* assays. In a similar fashion to QSSM 1112, farnesol also exhibited a profound concentration-dependent inhibition on filamentous growth of *C. albicans*. At the lowest concentration of 32 µg/ml, the farnesol has shown complete hyphal inhibition (0% hyphal) (**Figure 7C**). Although the influence of farnesol on the *C. albicans* dimorphic switching has already been demonstrated, the exact concentration required for yeast-to-hyphal inhibition varied from study to study owing to multiple associated factors. Mosel et al. (2005) reported that the concentration of farnesol required for the inhibition of yeast-to-hyphal transition has varied depending on the medium (Mosel et al., 2005). They found that 2 µM (0.44 µg/ml) and 250 µM (55.29 µg/ml) of farnesol were required to inhibit 50% of *C. albicans* hyphal formation in defined media and serum-containing media, respectively (Mosel et al., 2005). However, our study demonstrated that 32 µg/ml of farnesol was potent enough to inhibit the filamentation of *C. albicans* in both spider medium and serum. To further confirm the propensity of QSSM 1112 in inhibiting the *C. albicans* dimorphic behavior, solid spider agar assay was done. For that, overnight *C. albicans* culture was spotted on the spider agar medium supplemented with and without QSSM 1112 at its HIC (**Figure 7B**). After 2 days of incubation, the agar plates were directly visualized under the light microscope to observe the change in colony morphology upon QSSM 1112 treatment. The micrograph of untreated control has shown rough colony morphology with densely elongated hyphal cells. At the same time, the micrographs of QSSM 1112 and farnesol-treated cells have portrayed smooth colony morphology with abridged hyphal formation. In addition, after 7 days of incubation, the agar plates were documented using a gel documentation system (**Figure 7C**). The results signified the remarkable reduction of hyphal protrusion manifested with QSSM 1112 compared with the untreated control.

The hyphal inhibitory effect of farnesol was better than that of QSSM 1112, as it exhibited a substantial impact even at a very low concentration, i.e., even 2 µg/ml with 25% of hyphal inhibition. In contrast, QSSM 1112 at 2 µg/ml displayed a compactly elongated hyphal formation similar to that of control.

### 3.8 QSSM 1112 (at Hyphal Inhibitory Concentration) Averts *Candida albicans* Germ-Tube Formation

**Figure 8:**
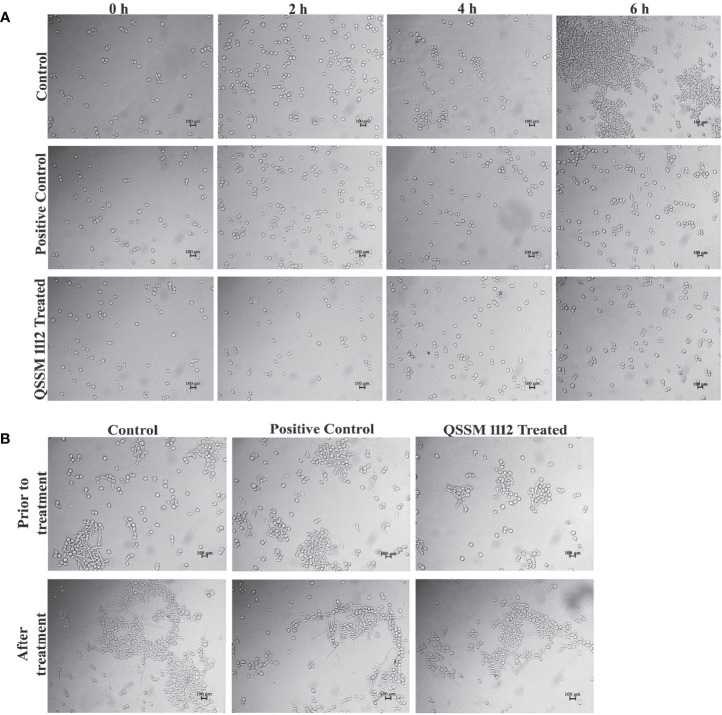
**(A)** The influence of QSSM 1112 (at HIC) and farnesol on *Candida albicans* germ-tube formation at various time intervals (0–6 h). The QSSM 1112 manifested into the FBS along with the *C. albicans* cells. The micrograph evidently demonstrates the blockage of germ-tube formation in *C. albicans* cells treated with QSSM 1112 compared with that of untreated control cells (which encompasses classic badminton structures, i.e., true germ-tubes of *C. albicans*). **(B)** The impact of QSSM 1112 (at HIC) and farnesol on preformed germ-tube formation. There is no significant difference between untreated control and QSSM 1112- and farnesol-treated cells. FBS, fetal bovine serum; HIC, hyphal inhibitory concentration.

The dimorphic growth of *C. albicans* is closely linked with the thin filamentous outgrowth extending from the blastospore known as “germ-tube.” The extended germ-tube later develops into true hyphae. Hence, the efficacy of QSSM 1112 (HIC) on *C. albicans* germ-tube formation was evaluated by culturing the *C. albicans* cells in FBS. In parallel, the effect of farnesol on germ-tube formation was evaluated for comparative purposes. The *C. albicans* cells were imaged in different time intervals (from 0 to 6 h) for comparison of germ-tube formation with and without QSSM 1112. As can be seen in **Figure 8A**, both QSSM 1112-treated and untreated control cells appeared as yeast form at the 0-h time interval. With the increase in time, few numbers of cells in untreated control samples showcased budding yeast morphology (a thin filamentous tube extending from yeast), and the true germ-tube germination was observed in the control sample at the time interval of 6 h. However, the cells treated with QSSM 1112 and farnesol (at HIC) portrayed complete spread of yeast cells, lacking the classic characteristics of hyphal elongation morphologies, *viz*., budding yeast followed by badminton shaped germ-tubes. This inevitably reinforces the data of *in vitro* liquid and solid antihyphal assays, which in turn signifies that QSSM 1112 followed the same mode of hyphal inhibitory action as that of *C. albicans* QS signaling molecule farnesol. In line with the present study, a similar kind of bacterial QSMs mediated germ-tube inhibition was observed by Zhang et al. (2011), wherein the two DSFs, i.e., *cis*- and *trans*-2-dodecenoic acid, have arrested the *C. albicans* cells from undergoing germ-tube induction (Zhang et al., 2011).

In most of the cases, the antihyphal inhibitors not only halt the germ-tube germination but also have the capability to revert the preformed germ-tube into yeast form. In order to know whether the QSSM 1112 exposure has any effect on preformed germ-tube, QSSM 1112 was added to wells that had preexisting *C. albicans* germ-tube. After 5-h time exposure, the preformed germ-tube cells were visualized under a light microscope. As can be seen from **Figure 8B**, no considerable change was observed in the germ-tube formation of both QSSM 1112-treated and untreated control cells. It was also observed that the positive control farnesol also had no effect; it neither reverted nor destroyed the germ-tube cells. This further confirms that QSSM 1112 and farnesol are ably averting *C. albicans* dimorphism before germination, whereas they could not have any effect on *C. albicans* cells that had germinated already. This result is in agreement with the observation of Mosel et al. where the farnesol appears to be ineffective on preexisting hyphae even at a very high concentration (Mosel et al., 2005). Although both farnesol and QSSM 1112 did not effectively inhibit preformed germ-tube formation, it has been reported that exposure with farnesol could sensitize the drug-resistant *C. albicans* to antifungal treatment. With the use of this same principle, several studies also demonstrated the synergistic combinatorial effect of farnesol over the conventional antifungals (amphotericin B, fluconazole, and micafungin) against *C. albicans* biofilm formation. In this way, QSSM also could be used in synergistic therapy to enhance the efficacy of a single dose.

### 3.9 Fluorescence Microscopic Analysis Reinforces the QSSM 1112-Mediated Hyphae and Germ-Tube Inhibition

**Figure 9:**
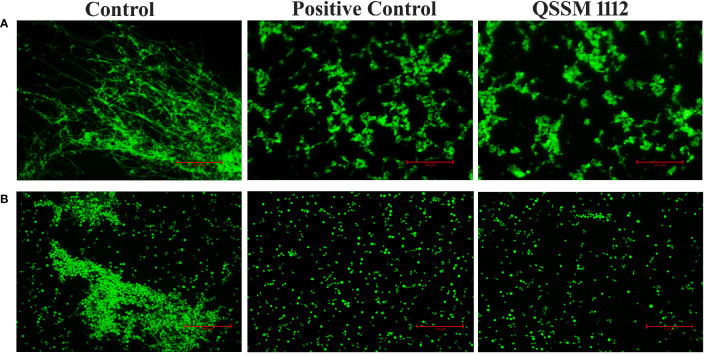
Fluorescence microscopic observation showcases the inhibitory potential of QSSM 1112 on **(A)** yeast-to-hyphal transition **(B)** germ-tube formation of *Candida albicans*.

In order to further reinforce the data obtained from a light microscopic analysis, fluorescence microscopic analysis was carried out. As seen in **Figures 9A, B**, QSSM 1112 is sufficient enough to inhibit the germ-tube and hyphal formation in *C. albicans*. Through *in vitro* validation of *in silico* results, the present investigation stands as a “proof of concept” for the reliability of *in silico*-based drug prediction in the drug development process.

## Conclusion

The current study investigates the antihyphal efficacy of bacterial QSMs through *in silico* and *in vitro* analyses against *C. albicans* dimorphic switching. Results of initial virtual screening analysis revealed hyphal inhibitory potential of the two DKPs, *viz*., QSSM 1112 and QSSM 1157, against CYCc and RAS1, respectively. Further, 50-ns MD simulation demonstrated the stable binding of ligands to target proteins with less conformational fluctuation and greater affinity. In addition, the pharmacokinetics and toxicity prediction revealed that the identified QSM hyphal inhibitors are safe for biological use. These QSMs displayed better lipophilicity and water solubility scores, lesser toxicity, and no BBB permeation. Further, *in vitro* investigations such as liquid and solid antihyphal assays and germ-tube inhibition assays unequivocally established QSSM 1112 as a promising hyphal inhibitor. On the whole, through *in silico* and *in vitro* analyses, the present study opens up new avenues for considering the bacterial QSMs as potential inhibitors against fungi especially *C. albicans*. This study also highlights that the antagonistic interaction between bacteria and fungi can be utilized as a tool to overcome certain fungal infections.

## Data Availability Statement

The original contributions presented in the study are included in the article/**Supplementary Material**. Further inquiries can be directed to the corresponding authors.

## Author Contributions

RJ designed the research idea, analyzed the data, carried out *in vitro* analysis and result interpretation, and wrote the original draft. NH carried out virtual screening, molecular docking and simulation analysis, and *in silico* result interpretation and prepared the figures. SG designed the research idea; performed the formal analysis, funding acquisition, investigation, administration, and validation; and critically revised the original draft. SP contributed to the conceptualization, supervision, funding acquisition, and critical revision of the original draft. All authors contributed to the article and approved the submitted version.

## Funding

SG gratefully acknowledges DST-SERB-EEQ Project Grant (File No.:EEQ/2020/000288). The authors thankfully acknowledge the financial support rendered by RUSA 2.0 [F.24-51/2014-U, Policy (TN Multi-Gen), Department of Education, Government of India].

## Conflict of Interest

The authors declare that the research was conducted in the absence of any commercial or financial relationships that could be construed as a potential conflict of interest.

## Publisher’s Note

All claims expressed in this article are solely those of the authors and do not necessarily represent those of their affiliated organizations, or those of the publisher, the editors and the reviewers. Any product that may be evaluated in this article, or claim that may be made by its manufacturer, is not guaranteed or endorsed by the publisher.

## Supplementary Material

Supplementary Table 1 - Physicochemical parameters, lipophilicity and solubility of QSSM 1157 & QSSM 1112.

## References

[B1] Abdulazeez S. (2019). Molecular Simulation Studies on B-Cell Lymphoma/Leukaemia 11A (BCL11A). Am. J. Transl. Res. 11 (6), 3689–3697. 31312380 PMC6614651

[B2] Baell J. B. Nissink J. W. M. (2018). Seven Year Itch: Pan-Assay Interference Compounds (PAINS) in 2017-Utility and Limitations. ACS Chem. Biol. 13 (1), 36–44. doi: 10.1021/acschembio.7b00903 29202222 PMC5778390

[B3] Banerjee P. Eckert A. O. Schrey A. K. Preissner R. (2018). ProTox-II: A Webserver for the Prediction of Toxicity of Chemicals. Nucleic Acids Res. 46 (W1), W257–W263. doi: 10.1093/nar/gky318 29718510 PMC6031011

[B4] Banerjee M. Lazzell A. L. Romo J. A. Lopez-Ribot J. L. Kadosh D. (2019). Filamentation Is Associated With Reduced Pathogenicity of Multiple Non-Albicans Candida Species. Msphere 4 (5), e00656–19. doi: 10.1128/mSphere.00656-19 PMC6796982 31619502

[B5] Barnes J. A. Williams I. H. (1996). Quantum Mechanical/Molecular Mechanical Approaches to Transition State Structure: Mechanism of Sialidase Action. Biochem. Soc. Trans. 24 (1), 263–268. doi: 10.1042/bst0240263 8674682

[B6] Bellezza I. Peirce M. J. Minelli A. (2014). Cyclic Dipeptides: From Bugs to Brain. Trends Mol. Med. 20 (10), 551–558. doi: 10.1016/j.molmed.2014.08.003 25217340 10.1016/j.molmed.2014.08.003

[B7] Boon C. Deng Y. Wang L. H. He Y. Xu J. L. Fan Y.. (2008). A Novel DSF-Like Signal From Burkholderia Cenocepacia Interferes With Candida Albicans Morphological Transition. ISME J. 2 (1), 27–36. doi: 10.1038/ismej.2007.76 18049456

[B8] Boutet E. Lieberherr D. Tognolli M. Schneider M. Bansal P. Bridge A. J.. (2016). UniProtKB/Swiss-Prot, the Manually Annotated Section of the UniProt KnowledgeBase: How to Use the Entry View. Methods Mol. Biol. 1374, 23–54. doi: 10.1007/978-1-4939-3167-5_2 26519399

[B9] Calderone R. A. Fonzi W. A. (2001). Virulence Factors of Candida Albicans. Trends Microbiol. 9 (7), 327–335. doi: 10.1016/s0966-842x(01)02094-7 11435107

[B10] Charro N. Mota L. J. (2015). Approaches Targeting the Type III Secretion System to Treat or Prevent Bacterial Infections. Expert Opin. Drug Discov. 10 (4), 373–387. doi: 10.1517/17460441.2015.1019860 25727140

[B11] Chen S. Xia J. Li C. Zuo L. Wei X. (2018). The Possible Molecular Mechanisms of Farnesol on the Antifungal Resistance of C. Albicans Biofilms: The Regulation of CYR1 and PDE2. BMC Microbiol. 18 (1), 203. doi: 10.1186/s12866-018-1344-z 30509171 PMC6278051

[B12] Dadgostar P. (2019). Antimicrobial Resistance: Implications and Costs. Infect. Drug Resist. 12, 3903–3910. doi: 10.2147/Idr.S234610 31908502 PMC6929930

[B13] Davis-Hanna A. Piispanen A. E. Stateva L. I. Hogan D. A. (2008). Farnesol and Dodecanol Effects on the Candida Albicans Ras1-cAMP Signalling Pathway and the Regulation of Morphogenesis. Mol. Microbiol. 67 (1), 47–62. doi: 10.1111/j.1365-2958.2007.06013.x 18078440 PMC3782305

[B14] de Carvalho M. P. Abraham W. R. (2012). Antimicrobial and Biofilm Inhibiting Diketopiperazines. Curr. Med. Chem. 19 (21), 3564–3577. doi: 10.2174/092986712801323243 22709011

[B15] Fiser A. (2010). Template-Based Protein Structure Modeling. Methods Mol. Biol. 673, 73–94. doi: 10.1007/978-1-60761-842-3_6 20835794 PMC4108304

[B16] Garcia L. G. S. da Rocha M. G. Lima L. R. Cunha A. P. de Oliveira J. S. de Andrade A. R. C.. (2021). Essential Oils Encapsulated in Chitosan Microparticles Against Candida Albicans Biofilms. Int. J. Biol. Macromol. 166, 621–632. doi: 10.1016/j.ijbiomac.2020.10.220 33137389

[B17] Gowrishankar S. Kamaladevi A. Balamurugan K. Pandian S. K. (2016a). *In Vitro* and *In Vivo* Biofilm Characterization of Methicillin-Resistant Staphylococcus Aureus From Patients Associated With Pharyngitis Infection. BioMed. Res. Int. 2016, 1289157. doi: 10.1155/2016/1289157 27761465 PMC5059529

[B18] Gowrishankar S. Pandian S. K. (2017). Modulation of Staphylococcus Epidermidis (RP62A) Extracellular Polymeric Layer by Marine Cyclic Dipeptide-Cyclo(L-Leucyl-L-Prolyl) Thwarts Biofilm Formation. Biochim. Biophys. Acta Biomembr. 1859 (7), 1254–1262. doi: 10.1016/j.bbamem.2017.04.009 28414038

[B19] Gowrishankar S. Pandian S. K. Balasubramaniam B. Balamurugan K. (2019). Quorum Quelling Efficacy of Marine Cyclic Dipeptide -Cyclo(L-Leucyl-L-Prolyl) Against the Uropathogen Serratia Marcescens. Food Chem. Toxicol. 123, 326–336. doi: 10.1016/j.fct.2018.11.013 30419322

[B20] Gowrishankar S. Poornima B. Pandian S. K. (2014). Inhibitory Efficacy of Cyclo(L-Leucyl-L-Prolyl) From Mangrove Rhizosphere Bacterium-Bacillus Amyloliquefaciens (MMS-50) Toward Cariogenic Properties of Streptococcus Mutans. Res. Microbiol. 165 (4), 278–289. doi: 10.1016/j.resmic.2014.03.004 24698790

[B21] Gowrishankar S. Sivaranjani M. Kamaladevi A. Ravi A. V. Balamurugan K. Karutha Pandian S. (2016b). Cyclic Dipeptide Cyclo(L-Leucyl-L-Prolyl) From Marine Bacillus Amyloliquefaciens Mitigates Biofilm Formation and Virulence in Listeria Monocytogenes. Pathog. Dis. 74 (4), ftw017. doi: 10.1093/femspd/ftw017 26945590

[B22] Grainha T. Jorge P. Alves D. Lopes S. P. Pereira M. O. (2020). Unraveling Pseudomonas Aeruginosa and Candida Albicans Communication in Coinfection Scenarios: Insights Through Network Analysis. Front. Cell. Infect. Microbiol. 10, 550505. doi: 10.3389/fcimb.2020.550505 33262953 PMC7686562

[B23] Hall R. A. Turner K. J. Chaloupka J. Cottier F. De Sordi L. Sanglard D.. (2011). The Quorum-Sensing Molecules Farnesol/Homoserine Lactone and Dodecanol Operate *via* Distinct Modes of Action in Candida Albicans. Eukaryot Cell 10 (8), 1034–1042. doi: 10.1128/EC.05060-11 21666074 PMC3165441

[B24] Hogan D. A. Vik A. Kolter R. (2004). A Pseudomonas Aeruginosa Quorum-Sensing Molecule Influences Candida Albicans Morphology. Mol. Microbiol. 54 (5), 1212–1223. doi: 10.1111/j.1365-2958.2004.04349.x 15554963

[B25] Hornby J. M. Jensen E. C. Lisec A. D. Tasto J. J. Jahnke B. Shoemaker R.. (2001). Quorum Sensing in the Dimorphic Fungus Candida Albicans is Mediated by Farnesol. Appl. Environ. Microbiol. 67 (7), 2982–2992. doi: 10.1128/Aem.67.7.2982-2992.2001 11425711 PMC92970

[B26] Human Z. R. Moon K. Bae M. de Beer Z. W. Cha S. Wingfield M. J.. (2016). Antifungal Streptomyces Spp. Associated With the Infructescences of Protea Spp. In South Africa. Front. Microbiol. 7, 1657. doi: 10.3389/fmicb.2016.01657 27853450 PMC5090004

[B27] Jabra-Rizk M. A. Meiller T. F. James C. E. Shirtliff M. E. (2006). Effect of Farnesol on Staphylococcus Aureus Biofilm Formation and Antimicrobial Susceptibility. Antimicrobial. Agents Chemother. 50 (4), 1463–1469. doi: 10.1128/Aac.50.4.1463-1469.2006 PMC1426993 16569866

[B28] Jha A. Kumar A. (2018). Deciphering the Role of Sodium Lignosulfonate Against Candida Spp. As Persuasive Anticandidal Agent. Int. J. Biol. Macromol. 107 (Pt A), 1212–1219. doi: 10.1016/j.ijbiomac.2017.09.102 28962848

[B29] Katragkou A. McCarthy M. Alexander E. L. Antachopoulos C. Meletiadis J. Jabra-Rizk M. A.. (2015). *In Vitro* Interactions Between Farnesol and Fluconazole, Amphotericin B or Micafungin Against *Candida Albicans* Biofilms. J. Antimicrobial. Chemother. 70, no. 2, 470–478. doi: 10.1093/jac/dku374 PMC4351386 25288679

[B30] Laskowski R. A. Chistyakov V. V. Thornton J. M. (2005). PDBsum More: New Summaries and Analyses of the Known 3D Structures of Proteins and Nucleic Acids. Nucleic Acids Res. 33, D266–D268. doi: 10.1093/nar/gki001 15608193 PMC539955

[B31] Lee J. H. Kim Y. G. Khadke S. K. Lee J. (2020). Antibiofilm and Antifungal Activities of Medium-Chain Fatty Acids Against Candida Albicans *via* Mimicking of the Quorum-Sensing Molecule Farnesol. Microbial. Biotechnol. doi: 10.1111/1751-7915.13710 PMC8313291 33252828

[B32] Lee S. K. Lee I. H. Kim H. J. Chang G. S. Chung J. E. No K. T. (2003). “The PreADME Approach: Web-Based Program for Rapid Prediction of Physico-Chemical, Drug Absorption and Drug-Like Properties,” in EuroQSAR 2002 Designing Drugs and Crop Protectants: Processes, Problems and Solutions 4 (21), 1353–1366.

[B33] Li C. Xu Z. Liu S. Huang R. Duan W. Wei X. (2021). *In Vivo* Antifungal Activity of Farnesol Combined With Antifungal Drugs on Oral Mucosal Candidiasis in Mice. Biofouling 15, 1–12. doi: 10.1080/08927014.2020.1869725 34579611

[B34] Li X. Quan C. S. Fan S. D. (2007). Antifungal Activity of a Novel Compound from *Burkholderia cepacia* Against Plant Pathogenic Fungi. Lett. Appl. Microbiol. 45 (5), 508–514. doi: 10.1111/j.1472-765X.2007.02221.x 17958556

[B35] Llor C. Bjerrum L. (2014). Antimicrobial Resistance: Risk Associated With Antibiotic Overuse and Initiatives to Reduce the Problem. Ther. Adv. Drug Saf. 5 (6), 229–241. doi: 10.1177/2042098614554919 25436105 PMC4232501

[B36] Lynch T. Price A. (2007). The Effect of Cytochrome P450 Metabolism on Drug Response, Interactions, and Adverse Effects. Am. Fam. Phys. 76 (3), 391–396. 17708140

[B37] Maia E. H. B. Assis L. C. de Oliveira T. A. da Silva A. M. Taranto A. G. (2020). Structure-Based Virtual Screening: From Classical to Artificial Intelligence. Front. Chem. 8, 343. doi: 10.3389/fchem.2020.00343 32411671 PMC7200080

[B38] Manoharan R. K. Lee J. H. Lee J. (2017). Antibiofilm and Antihyphal Activities of Cedar Leaf Essential Oil, Camphor, and Fenchone Derivatives Against Candida Albicans. Front. Microbiol. 8, 1476. doi: 10.3389/fmicb.2017.01476 28824600 PMC5541024

[B39] Mosel D. D. Dumitru R. Hornby J. M. Atkin A. L. Nickerson K. W. (2005). Farnesol Concentrations Required to Block Germ Tube Formation in Candida Albicans in the Presence and Absence of Serum. Appl. Environ. Microbiol. 71 (8), 4938–4940. doi: 10.1128/AEM.71.8.4938-4940.2005 16085901 PMC1183276

[B40] Nickerson K. W. Atkin A. L. (2017). Deciphering Fungal Dimorphism: Farnesol’s Unanswered Questions. Mol. Microbiol. 103 (4), 567–575. doi: 10.1111/mmi.13601 27987234

[B41] Opo F. Rahman M. M. Ahammad F. Ahmed I. Bhuiyan M. A. Asiri A. M. (2021). Structure Based Pharmacophore Modeling, Virtual Screening, Molecular Docking and ADMET Approaches for Identification of Natural Anti-Cancer Agents Targeting XIAP Protein. Sci. Rep. 11 (1), 4049. doi: 10.1038/s41598-021-83626-x 33603068 PMC7892887

[B42] Priya A. Pandian S. K. (2020). Piperine Impedes Biofilm Formation and Hyphal Morphogenesis of Candida Albicans. Front. Microbiol. 11, 756. doi: 10.3389/fmicb.2020.00756 32477284 PMC7237707

[B43] Raies A. B. Bajic V. B. (2016). *In Silico* Toxicology: Computational Methods for the Prediction of Chemical Toxicity. Wiley Interdiscip. Rev. Comput. Mol. Sci. 6 (2), 147–172. doi: 10.1002/wcms.1240 27066112 PMC4785608

[B44] Rajput A. Kaur K. Kumar M. (2016). SigMol: Repertoire of Quorum Sensing Signaling Molecules in Prokaryotes. Nucleic Acids Res. 44 (D1), D634–D639. doi: 10.1093/nar/gkv1076 26490957 PMC4702795

[B45] Ramage G. Saville S. P. Wickes B. L. Lopez-Ribot J. L. (2002). Inhibition of Candida Albicans Biofilm Formation by Farnesol, a Quorum-Sensing Molecule. Appl. Environ. Microbiol. 68 (11), 5459–5463. doi: 10.1128/Aem.68.11.5459-5463.2002 12406738 PMC129887

[B46] Rhee K. H. (2004). Cyclic Dipeptides Exhibit Synergistic, Broad Spectrum Antimicrobial Effects and Have Anti-Mutagenic Properties. Int. J. Antimicrob. Agents 24 (5), 423–427. doi: 10.1016/j.ijantimicag.2004.05.005 15519471

[B47] Rodrigues A. M. X. Costa R. K. M. Machado R. S. Gutierrez S. J. C. Lima F. D. C. A. de Oliveira A. P. (2020). *In-Silico* Studies of Riparin B in the Design of Drugs: Physicochemical, Pharmacokinetic and Pharmacodynamic Parameters. bioRxiv. doi: 10.1101/2020.04.24.059626

[B48] Roemer T. Krysan D. J. (2014). Antifungal Drug Development: Challenges, Unmet Clinical Needs, and New Approaches. Cold Spring Harbor Perspect. Med. 4 (5), a019703. doi: 10.1101/cshperspect.a019703 PMC3996373 24789878

[B49] Rolta R. Yadav R. Salaria D. Trivedi S. Imran M. Sourirajan A.. (2020). *In Silico* Screening of Hundred Phytocompounds of Ten Medicinal Plants as Potential Inhibitors of Nucleocapsid Phosphoprotein of COVID-19: An Approach to Prevent Virus Assembly. J. Biomol. Struct. Dyn. 39 (18), 7017–7034. doi: 10.1080/07391102.2020.1804457 32851912 PMC7484575

[B50] Sato T. Watanabe T. Mikami T. Matsumoto T. (2004). Farnesol, a Morphogenetic Autoregulatory Substance in the Dimorphic Fungus Candida Albicans, Inhibits Hyphae Growth Through Suppression of a Mitogen-Activated Protein Kinase Cascade. Biol. Pharm. Bull. 27 (5), 751–752. doi: 10.1248/bpb.27.751 15133261

[B51] Satyanarayana S. D. V. Krishna M. S. R. Pavan Kumar P. Jeereddy S. (2018). *In Silico* Structural Homology Modeling of Nif A Protein of Rhizobial Strains in Selective Legume Plants. J. Genet. Eng. Biotechnol. 16 (2), 731–737. doi: 10.1016/j.jgeb.2018.06.006 30733794 PMC6353771

[B52] Schuttelkopf A. W. van Aalten D. M. F. (2004). PRODRG: A Tool for High-Throughput Crystallography of Protein-Ligand Complexes. Acta Crystallogr. Sect. D-Struct. Biol. 60, 1355–1363. doi: 10.1107/S0907444904011679 15272157

[B53] Shchepin R. Hornby J. M. Burger E. Niessen T. Dussault P. Nickerson K. W. (2003). Quorum Sensing in Candida Albicans: Probing Farnesol’s Mode of Action With 40 Natural and Synthetic Farnesol Analogs. Chem. Biol. 10 (8), 743–750. doi: 10.1016/S1074-5521(03)00158-3 12954333

[B54] Shima S. MATSUOKA H. IWAMOTO T. SAKAI H. (1984). Antimicrobial Action of ϵ-Poly-L-Lysine. J. Antibiot. 37 (11), 1449–1455. doi: 10.7164/antibiotics.37.1449 6392269

[B55] Singh M. P. Petersen P. J. Weiss W. J. Kong F. Greenstein M. (2000). Saccharomicins, Novel Heptadecaglycoside Antibiotics Produced by Saccharothrix Espanaensis: Antibacterial and Mechanistic Activities. Antimicrob. Agents Chemother. 44 (8), 2154–2159. doi: 10.1128/AAC.44.8.2154-2159.2000 10898690 PMC90028

[B56] Sobolev O. V. Afonine P. V. Moriarty N. W. Hekkelman M. L. Joosten R. P. Perrakis A.. (2020). A Global Ramachandran Score Identifies Protein Structures With Unlikely Stereochemistry. Structure 28 1249-1258 (11), e1242. doi: 10.1016/j.str.2020.08.005 PMC7642142 32857966

[B57] Srivastava V. Dubey A. K. (2016). Anti-Biofilm Activity of the Metabolites of *Streptomyces Chrestomyceticus* Strain ADP4 Against *Candida Albicans* . J. Biosci. Bioeng. 122 (4), 434–440. doi: 10.1016/j.jbiosc.2016.03.013 27117484

[B58] Tuntland T. Ethell B. Kosaka T. Blasco F. Zang R. X. Jain M.. (2014). Implementation of Pharmacokinetic and Pharmacodynamic Strategies in Early Research Phases of Drug Discovery and Development at Novartis Institute of Biomedical Research. Front. Pharmacol. 5, 174. doi: 10.3389/fphar.2014.00174 25120485 PMC4112793

[B59] Vyas V. K. Ukawala R. D. Ghate M. Chintha C. (2012). Homology Modeling a Fast Tool for Drug Discovery: Current Perspectives. Indian J. Pharm. Sci. 74 (1), 1–17. doi: 10.4103/0250-474x.102537 23204616 PMC3507339

[B60] Wang Y. (2013). Fungal Adenylyl Cyclase Acts As a Signal Sensor and Integrator and Plays a Central Role in Interaction With Bacteria. PloS Pathog. 9 (10), e1003612. doi: 10.1371/journal.ppat.1003612 24130478 PMC3795026

[B61] Yan P. S. Song Y. Sakuno E. Nakajima H. Nakagawa H. Yabe K. (2004). Cyclo(L-Leucyl-L-Prolyl) Produced by Achromobacter Xylosoxidans Inhibits Aflatoxin Production by Aspergillus Parasiticus. Appl. Environ. Microbiol. 70 (12), 7466–7473. doi: 10.1128/AEM.70.12.7466-7473.2004 15574949 PMC535151

[B62] Yin M. Yan Y. Lohman J. R. Huang S. X. Ma M. Zhao G. R.. (2014). Cycloheximide and Actiphenol Production in Streptomyces Sp. YIM56141 Governed by Single Biosynthetic Machinery Featuring an Acyltransferase-Less Type I Polyketide Synthase. Org. Lett. 16 (11), 3072–3075. doi: 10.1021/ol501179w 24815182 PMC4051428

[B63] Zhang Y. Cai C. Yang Y. Weng L. Wang L. (2011). Blocking of Candida Albicans Biofilm Formation by Cis-2-Dodecenoic Acid and Trans-2-Dodecenoic Acid. J. Med. Microbiol. 60 (Pt 11), 1643–1650. doi: 10.1099/jmm.0.029058-0 21778264

