# Supplementary material for: Bacterial Quorum-Sensing Molecules as Promising Natural Inhibitors of Candida albicans Virulence Dimorphism: An In Silico and In Vitro Study
Source: Front Cell Infect Microbiol. 2021 Dec 3;11:781790. doi: 10.3389/fcimb.2021.781790 (PMC8677694; doi:10.3389/fcimb.2021.781790)
Supplement: Supplementary Table 1 — - Physicochemical parameters, lipophilicity and solubility of QSSM 1157 & QSSM 1112. [file Table_1.doc]

| **Various Parameters** | **QSSM 1112** | **QSSM 1157** |
| --- | --- | --- |
| **Physicochemical Properties** | | |
| Formula | C11H2N2O3 | C15H2N2O2 |
| Molecular weight | 210.15 g/mol | 242.19 g/mol |
| No of heavy atoms | 16 | 19 |
| No of aromatic heavy atoms | 5 | 6 |
| Fraction Csp3 | 0.45 | 0.33 |
| No of rotatable bonds | 2 | 3 |
| No of H-bond acceptors | 3 | 2 |
| No of H-bond donors | 2 | 2 |
| Molar Refractivity | 51.5 | 64.96 |
| [TPSA](http://www.swissadme.ch/index.php) | 71.33 Å² | 58.20 Å² |
| **Lipophilicity** | | |
| [Log Po/w (iLOGP)](http://www.swissadme.ch/index.php) | 0 | 0 |
| [Log Po/w (XLOGP3)](http://www.swissadme.ch/index.php) | 1.33 | 2.31 |
| [Log Po/w (WLOGP)](http://www.swissadme.ch/index.php) | -1.13 | -1.89 |
| [Log Po/w (MLOGP)](http://www.swissadme.ch/index.php) | 0.52 | 1.36 |
| [Log Po/w (SILICOS-IT)](http://www.swissadme.ch/index.php) | 0.67 | 2.55 |
| [Consensus Log Po/w](http://www.swissadme.ch/index.php) | 0.28 | 0.86 |
| **Water Solubility** | | |
| [Log S (ESOL)](http://www.swissadme.ch/index.php) | -2.08 | -2.83 |
| Solubility | 1.75e+00 mg/ml; 8.32e-03 mol/l | 3.56e-01 mg/ml; 1.47e-03 mol/l |
| [Class](http://www.swissadme.ch/index.php) | Soluble | Soluble |
| [Log S (Ali)](http://www.swissadme.ch/index.php) | -2.43 | -3.17 |
| Solubility | 7.82e-01 mg/ml ; 3.72e-03 mol/l | 1.64e-01 mg/ml ; 6.75e-04 mol/l |
| [Class](http://www.swissadme.ch/index.php) | Soluble | Soluble |
| [Log S (SILICOS-IT)](http://www.swissadme.ch/index.php) | -1.87 | -4.42 |
| Solubility | 2.82e+00 mg/ml ; 1.34e-02 mol/l | 9.25e-03 mg/ml ; 3.82e-05 mol/l |
| [Class](http://www.swissadme.ch/index.php) | Soluble | Moderately soluble |
